# Quantification of the calcium signaling deficit in muscles devoid of triadin

**DOI:** 10.1371/journal.pone.0264146

**Published:** 2022-02-25

**Authors:** Carlo Manno, Eshwar Tammineni, Lourdes Figueroa, Isabelle Marty, Eduardo Ríos

**Affiliations:** 1 Department of Physiology and Biophysics, Rush University Medical Center, Chicago, Illinois, United States of America; 2 Grenoble-Institut des Neurosciences (INSERM U1216), La Tronche, France; Indiana University School of Medicine, UNITED STATES

## Abstract

Triadin, a protein of the sarcoplasmic reticulum (SR) of striated muscles, anchors the calcium-storing protein calsequestrin to calcium release RyR channels at the junction with t-tubules, and modulates these channels by conformational effects. Triadin ablation induces structural SR changes and alters the expression of other proteins. Here we quantify alterations of calcium signaling in single skeletal myofibers of constitutive triadin-null mice. We find higher resting cytosolic and lower SR-luminal [Ca^2+^], 40% lower calsequestrin expression, and more Ca_V_1.1, RyR1 and SERCA1. Despite the increased Ca_V_1.1, the mobile intramembrane charge was reduced by ~20% in Triadin-null fibers. The initial peak of calcium release flux by pulse depolarization was minimally altered in the null fibers (revealing an increase in peak calcium permeability). The “hump” phase that followed, attributable to calcium detaching from calsequestrin, was 25% lower, a smaller change than expected from the reduced calsequestrin content and calcium saturation. The exponential decay rate of calcium transients was 25% higher, consistent with the higher SERCA1 content. Recovery of calcium flux after a depleting depolarization was faster in triadin-null myofibers, consistent with the increased uptake rate and lower SR calsequestrin content. In sum, the triadin knockout determines an increased RyR1 channel openness, which depletes the SR, a substantial loss of calsequestrin and gains in other couplon proteins. Powerful functional compensations ensue: activation of SOCE that increases [Ca^2+^]_cyto_; increased SERCA1 activity, which limits the decrease in [Ca^2+^]_SR_ and a restoration of SR calcium storage of unknown substrate. Together, they effectively limit the functional loss in skeletal muscles.

## Introduction

In the excitation-contraction (EC) coupling process of striated muscles, action potentials command the transient release of Ca^2+^ into the myoplasm, enabling muscle contraction. The crucial device in this process is the couplon, a physical continuum of proteins that includes the dihydropyridine receptor (DHPR, Ca_V_1.1), the ryanodine receptor 1 (RyR1), FKBP12, junctophilin 1 (JPh1), stac3, junctin (Jct), triadin (Tr) and calsequestrin (Casq1), among other components [1, 2]. A number of myopathies have their origin in structural and functional alterations caused by mutations in proteins of the couplon [3].

Five couplon proteins: the α1 and *β*1*a* subunits of Ca_V_1.1, RyR1, JPh1, and Stac3, have been found essential for EC coupling [4]. The others accomplish tasks necessary for maintaining normal muscle development, function and structure. Triadin [5, 6], encoded by the *TRDN* gene alternatively spliced into 4 isoforms, is a single-pass transmembrane protein of the SR membrane [7, 8]. The full-length 95-kDa isoform is a component of the skeletal muscle couplon.

The role of this protein has been controversial since its first description; initially thought to be an essential relay between voltage and Ca^2+^ signaling for EC coupling [9], a consensus on its function as a modulator of the Ca^2+^ signal was implied by two review articles of 2009 [7, 10]. In the years since, roles in the development and maintenance of junctional structure have been reported [11–14]. The interaction region, first described as hyperreactive sulfhydryls localized on RyR and triadin, by Liu & Pessah, (1994) [15], later, the region responsible for triadin’s positive effect on RyR1 function [16] was identified. An additional molecular mechanism involving the destabilization of RyR1 activity by a decreased interaction with FKBP12 in triadin-null (Tr-null) myotubes, was proposed by Eltit *et al*. (2010, 2011) [17, 18].

The relevance of triadin was brought into sharper focus by studies in cardiac muscle. First came the demonstration of its role in maintenance of junctional structure, largely as a tether to keep the polymeric Ca^2+^-storage protein Casq1 [19] within the SR terminal cisternae (TC), a role thought to be shared with the structurally related junctin [20]. More recently, mutations in *TRDN* that lead to functional protein ablation in humans [21] based the recognition of a “triadin knockout syndrome”, with its own registry [22, 23]; these studies also installed as a question the surprising functional tolerance of skeletal muscle to triadin null mutations (e.g. Engel *et al*. (2017) [24]).

The consequences of triadin ablation should be due, at least in part, to the substantial concomitant reduction in content of Casq1, by ~40% in the case of skeletal muscle, observed in the different triadin knockout mouse models developed to date [11, 25]. Calsequestrin is the main Ca^2+^ binding protein of the SR [26]. Casq1 is estimated to store 75% of the releasable Ca^2+^ in the SR of skeletal muscle [27], hence a reduction in its content should directly affect the availability of calcium to signal for EC coupling. Additionally, the cardiac isoform of calsequestrin, Casq2, is known to contribute to the modulation of the open-close gating of the RyR2 channel [28, 29]. This role, demonstrated in bilayer studies (e.g., [30]), was confirmed for the skeletal muscle isoform by studies of Casq1-null skeletal muscle [31, 32] that revealed a “Ca^2+^-sensing” function of the protein, which determines channel closing when [Ca^2+^]_SR_ falls below a certain level [32]. Triadin, physically bound to RyR and calsequestrin, is believed to constitute the conformational link that mediates the storage protein’s modulatory effects on gating of Ca^2+^ release.

Previously, our work identified a kinetic feature, named the “hump”, in the waveform of Ca^2+^ release flux elicited in skeletal myofibers by voltage-clamp stimulation [33, 34]. The hump operates as a signature of the reversible Ca^2+^ storage function of Casq1, as it is absent in Casq1-null muscle, is transiently disabled upon depletion of the SR calcium content and reappears as Casq1 recovers from depletion-induced depolymerization [35]. Isolation of the hump allows determination of a lower limit for the quantity of Ca^2+^ dissociated from Casq1 at any time. In the present study we applied these experimental tools to evaluate the quantitative deficit and qualitative modification of Ca^2+^ release function in murine skeletal muscle constitutively null for triadin. The expectation was that an accurate determination of loss or alteration of the component stored in murine Casq1 would help understand the mechanisms brought to play by the triadin deletion in animals and patients.

To interpret the results mechanistically, in terms of alterations of the main proteins and processes required for EC coupling, we quantified intramembrane charge movement (largely carried by Ca_V_1.1) and its voltage dependence; we also measured resting free [Ca^2+^] in both cytosol and SR lumen, evaluated Ca^2+^ release flux kinetically and calculated quantity of releasable Ca^2+^. Finally, we measured the expression density of Casq1 as well as structural parameters of SR terminal cisternae in living cells, in images of fluorescently tagged Casq1. The separation from wild-type values found in the calcium movements of triadin-null muscle could be assigned only in part to a reduction in SR Ca^2+^ content associated with the loss of Casq1. Other robust compensatory mechanisms are therefore exposed or implied by the present observations.

## Methods

### Ethical approval

Protocols on usage, care, transfection and killing of animals were approved by the Institutional Animal Care and Use Committee of Rush University, and were consistent with their ethical standards.

### Animals

Pan triadin-null (Tr-null) mice were derived by crossing individuals derived by reactivation (kindly authorized by Drs. C. Pérez and P.D. Allen) of frozen sperm from the Tr-null colony engineered by Shen *et al*. (2007) [25] with mice from the colony engineered by Oddoux *et al*. (2009) [11]. Both animal models were unintentionally crossed in the animal facility. The novel, C57BL/6 colony, is stable and its muscles are triadin-free. Wild type (WT) littermates from original breeding facility were not available for this study; WT mice of the strain C57BL/6 were obtained from Charles River Breeding Laboratories, Wilmington, MA.

### Solutions

**Normal Tyrode** (mM): 140 NaCl, 5 KCl, 2.5 CaCl_2_, 2 MgCl_2_, 10 HEPES, 10 glucose, pH was adjusted to 7.2 with NaOH. **External solution**: 140 TEA-CH_3_SO_3_, 1 CaCl_2_, 3.5 MgCl_2_, 10 HEPES, 1 4-aminopyridine, 0.5 CdCl_2_, 0.3 LaCl_3_, 0.001 TTX (citrate), 10 glucose, 0.025 *N*-benzyl-*p*-toluene sulphonamide (BTS), pH was adjusted to 7.2 with TEA-OH and osmolality to 320 mosmol kg^-1^ with TEA methanesulphonate. **Internal solutions** (mM): 120 K-L-glutamate, 5 BAPTA, 10 Trizma, 5 Na-ATP, 5 phosphocreatine Tris, 5.4 CaCl_2_, 7.19 MgCl_2_, 10 glucose, 100 nM free Ca^2+^, 0.05 Fluo-4 (pentapotassium salt).

### Preparation of plasmids

In most cases muscles were made to express either the biosensor D4cpV-Casq1, for intra-SR [Ca^2+^] measurements, or a variant, CFP-Casq1, to evaluate the pattern of Casq1 expression inside the lumen of SR. Plasmids were prepared as described by Sztretye et al., (2011*b*) [36]. Briefly, two D4cpV fusion plasmids were assembled: D4cpV-Casq1, combining cDNA of mouse Casq1 [37] with pBAD/D4cpV [38]. Transfection and isolation of FDB muscle followed methods of DiFranco *et al*. (2006) [39] and were used four days after electroporation.

### Resting calcium concentration in cytosol and sarcoplasmic reticulum

Resting cytosolic [Ca^2+^], [Ca^2+^]_cyto_, was monitored by SEER (Shifted Excitation and Emission Ratioing) of Indo-1 fluorescence [40], in FDB muscles. The dye was loaded by exposure of whole muscles to a 5 μM solution of its AM derivative for 30 min at room temperature. Isolated muscles were washed with fresh Tyrode solution and allowed to rest for 1 h before measurements. [Ca^2+^]_cyto_ was calculated (as described in Launikonis *et al*. (2005) [40]), from the ratio *R*(*x*,*y*,*t*) of fluorescence images *F*_1_ and *F*_2_ of Indo-1 excited at 351 and 364 nm and collected between 390 and 440 nm (*F*_1_) or between 465 and 535 nm (*F*_2_), by the equation:

$$[Ca^{2+}](x,y)=\gamma K_{D}\frac{R(x,y)-R_{min}}{R_{max}-R(x,y)}$$ (1)

Here γ is the ratio *F*_1_ of Ca^2+^-free dye /*F*_1_ Ca^2+^-saturated dye. *R*_*min*_ (0.23), *R*_*max*_ (4.06), and γ*K*_*D*_ (1.01 μM), were determined by calibrations *in situ*. The resting values reported are averages over the area of cytosol spanned by the image.

Luminal SR [Ca^2+^], [Ca^2+^]_SR_, was determined as described in Sztretye *et al*. (2011*b*) [36], from the FRET (Förster resonant energy transfer) ratio *R* of the biosensor D4cpV-Casq1, using Eq 1. Parameters, determined by calibrating the sensor *in situ*, were *R*_*min*_ (0.32), *R*_*max*_ (1.43), and γ*K*_*D*_ (222 μM). In all cases, *R* was calculated after subtraction of corresponding backgrounds measured with the laser off. (Note that, for simplicity, the symbol *R* is used for fluorescence ratios as well as quantity of calcium released.)

### Electrophysiology

6- to 12-week-old WT or Tr-null mice were euthanized by CO_2_ inhalation and subsequent cervical dislocation. FDB muscles were immediately removed and enzymatically treated with 2 mg/ml collagenase type I (Sigma, St. Louis, MO), in MEM-alpha media plus 10% fetal bovine serum (FBS) for 45 min at 37°C. Muscles were then washed and placed in Tyrode plus 10% FBS at 4°C for 2 h, then dissociated into single cells. The fibers used were carefully chosen to have similar shape and size. Fibers with apparent contractions or altered structure were excluded from our measurements.

Pipettes were pulled from borosilicate glass capillaries (Harvard Apparatus, Holliston, MA, USA) using a vertical micropipette puller (PC-10; Narishige, Amityville, NY, USA). The pipettes were heat-polished to a tip diameter of 3–4 μm. Myofibers were placed in “external solution”, patched near their center and clamped at −90 mV, using an Axopatch 200B amplifier (Axon Instruments, Foster City, CA, USA), for at least 20 min before the actual recording of Ca^2+^ transients. The clamped cells were stable, as ascertained by the stability of series resistance, linear capacitance, charging time constant and holding current.

Intramembranous gating charge displacements *Q*_*on*_ and *Q*_*off*_ were calculated as the time integral of the charge movement currents (*I*_*Q*_(*t*)). For *Q*_*on*_ the integral extended to the end of the pulse. For *Q*_*off*_, to 50 ms after the pulse. Similarly, the total quantity of released calcium *R*_*T*_, was calculated by integrating the Ca^2+^ release flux. The dependences of *Q* or *R* on test potential *V*, were fitted by a “Boltzmann” function of the type:

X(V)=Xmax(1+e−(V−V¯)κx) (2)

Where *X*(*V*) represents either *Q* or *R*_*T*_, and *X*_*max*_ is the maximum charge transferred or maximum total calcium released. $\bar{V}$ is the central voltage of the distribution and *κ* a “steepness” parameter, inversely proportional to the effective valence of the mobile charge or the charge activating Ca^2+^ release [41]. Data acquisition, pulse generation and synchronization with confocal imaging were implemented by two dedicated computers combining commercial and custom software.

### Imaging, cytosolic [Ca^2+^], Ca^2+^ release flux and released quantity

After 20 minutes of equilibration of pipette internal solution inside cells, fluorescence of Fluo-4 excited with 488 nm light was acquired in the range 500–545 nm by a confocal microscope (SP2 AOBS, Leica Microsystems, Exton, PA, USA). Fig 4*A* shows a line-scan image *F*(*x*,*t*). A summary time-dependent fluorescence *F*(*t*) was calculated by averaging over the *x* coordinate for the extent of the image. Cytosolic Ca^2+^ concentration [Ca^2+^]_cyto_(*t*) was calculated by

[Ca2+]cyto(t)=(F(t)−Fmin)koff+dFdt(Fmax−F(t))kon (3)

*F*_*max*_ and *F*_*min*_ are the fluorescence intensities at zero and saturating [Ca^2+^]. The kinetic values for Fluo-4 were determined elsewhere [33, 42], where the dissociation rate constant *k*_*off*_ was set at 0.175 ms^-1^ and the *k*_*d*_ at 0.74 μM.

Ca^2+^ release flux $\overset{˙}{R}(t)$ was derived from [Ca^2+^](*t*) by a simplified version of the “removal” method described previously [33, 43, 44], which involves assigning parameter values in a model of the removal of released Ca^2+^, so that the simulation fits the observed decay of cytosolic [Ca^2+^] simultaneously for several evoked transients. This procedure is hampered by lack of knowledge of multiple cellular processes involved in the evolution of [Ca^2+^]_cyto_, including the many molecules that bind Ca^2+^ and transport processes that move it. However, in the presence of a high concentration of a Ca^2+^ buffer, BAPTA in the present case, the incidence of the endogenous buffers becomes negligible [45] and the calculation of flux depends only on five parameters. (Please see an evaluation of the actual buffering power of the cytosol in the final section of Discussion.) Empirically, we found that the fitting of the *off* transient required by the procedure can be done allowing only one of these parameters to vary, namely, the rate of pump removal *k*_*uptake*_ for best fit varied between 3.5 and 7 ms^-1^. [Dye]_total_ and [BAPTA]_total_, were set to 0.5 of the concentration in the pipette for records that started after 30 min in the whole cell configuration. The kinetic constants of BAPTA: Ca^2+^ reaction were *k*_*on*_ = 1000 μM*s^-1^ and *k*_*off*_ = 200 s^-1^ [46]. That the calculation could be done with essentially the same parameter values for all myofibers endorses the comparison of fluxes, calculated thus, across myofibers and individual animals. On the other hand, and given the assumptions required, the accuracy of values derived with this method remains questionable.

The method also allows quantification of *R*(*t*), the quantity of Ca^2+^ released at time *t*, by integration of flux over time from the beginning of the pulse.


$$R(t)=\int_{0}^{t}\overset{˙}{R}(u)du$$ (4)

The net flux leaving the SR is calculated by subtracting the pump removal flux:

$${\overset{˙}{R}}_{net}(t)=\overset{˙}{R}(t)-k_{uptake}{\left[Ca^{2+}\right]}_{cyto}(t)$$ (5)

*R*_*net*_(*t*) the net quantity leaving the SR, is calculated as done for *R*(*t*), as the time integral of ${\overset{˙}{R}}_{net}(t)$ from the beginning of the pulse.

Fluxes and released quantities were evaluated at specific times or time ranges, for comparisons between WT and Tr-null muscles. The measured values include the early peak flux, which is also the absolute maximum, ${\dot{R}}_{P}$, and three quantities of Ca^2+^ released: the integral of flux during the initial “peak” stage, *R*_*P*_, the total released during the pulse, *R*_*T*_, and the quantity of Ca^2+^ released during the “hump” phase that follows the peak, *R*_*H*_. As a simplification we assumed that Ca^2+^ release consists of just the peak and hump stages, therefore *R*_*H*_ was calculated as *R*_*T*_−*R*_*P*_. These definitions are illustrated in Fig 4*B*. Of note: the integrals of flux are referred to as quantities, but have dimensions of concentration, because fluxes and quantities are calculated per unit volume of cytosol.

### Determination of myofiber type

Fiber types were identified by immunofluorescence imaging analysis using monoclonal antibodies specific to myosin heavy chain (MHC) isoforms attributed to each cell type.

FDB cross-sections were prepared as described by Beedle, (2016) [47]. Briefly, muscles were dissected and introduced into the Optimal Cutting Temperature (OCT) compound, and subsequently transferred into isopentane, previously cooled in liquid nitrogen. Cryopreserved samples were cut (Cryostat CM1850, Leica, Wetzlar, Germany), with section thickness of 10 *μm*. Frozen cross-sections were fixed in 4% paraformaldehyde (PFA) for 20 min and washed in phosphate buffered saline (PBS). Fixed cross-sections were incubated in 0.2% Triton X-100 (Sigma, St. Louis, MO), for 30 min, and then incubated in 5% goat serum (G9023, Sigma, St. Louis, MO), for 1 h. Incubation in primary antibodies occurred overnight at 4°C. Cross-sections were incubated in secondary antibodies for 2 h, at room temperature.

Primary antibodies (20 *μg*/*ml*): type I (DSHB BA-F8), type IIa (DSHB SC-71) and type IIx (DSHB 6H1). Secondary antibodies (10 *μg*/*ml*): type I (ThermoFisher A-21242), type IIa (Thermofisher A-21121) and type IIx (ThermoFisher A-21044). Signals were acquired under the “sequential” configuration, where the crosstalk between emission wavelengths was absent.

### Width of SR terminal cisternae

To evaluate the pattern of Casq1 expression inside the SR terminal cisternae (TC), cells expressing the fusion protein CFP-Casq1 were exposed for 30 min at room temperature to a Tyrode solution with 5 μM di-8-ANEPPS, a marker of surface and t tubule membranes. Imaging was done using a laser scanning confocal system with continuously adjustable excitation wavelength and high-sensitivity “HyD” hybrid detectors (Falcon SP8, Leica Microsystems, Wetzlar, Germany). Images were corrected for optical spread, either *in situ*, by a proprietary deconvolution method named “Lightning” (Leica), or a custom deconvolution method (implemented in the “Huygens” environment; SVI, Amsterdam), which uses the PSF (point spread function) of the microscope measured in our lab. A one-dimensional measure of the spatial extent of TC in the direction of the myofiber axis (*x* axis in images) was calculated as the average projection in the vertical axis of the joint span of the t tubule plus the two TC; this distance was defined as “Triad width”. When applying an all-neighbors correction procedure, the best correction is achieved near the middle of the stack. Hence, in these stacks of approximately 40 images, the measure of TC width was carried out on image 19, 20 or 21, after correction.

### Analysis of proteins

Total muscle homogenate was prepared from each animal in a similar manner as described by Oddoux *et al*. (2009) [11]. Briefly, *FDB* muscles from WT and Tr-null mice were dissected and chopped into small pieces in (mM): 200 sucrose, 0.4 CaCl_2_, 20 HEPES (pH = 7.4), containing 1:100 protease (P8340, Sigma, St. Louis, MO) and 1:100 phosphatase (SC-45044, Santa Cruz Biotechnology, Dallas, TX) inhibitor cocktails. Samples were further homogenized using a Polytron disrupter (Pro-01200, Pro Scientific, Oxford, CT). Protein content was quantified by the BCA protein assay (Thermo Fisher Scientific, Waltham, MA, USA).

**Fig 1:**
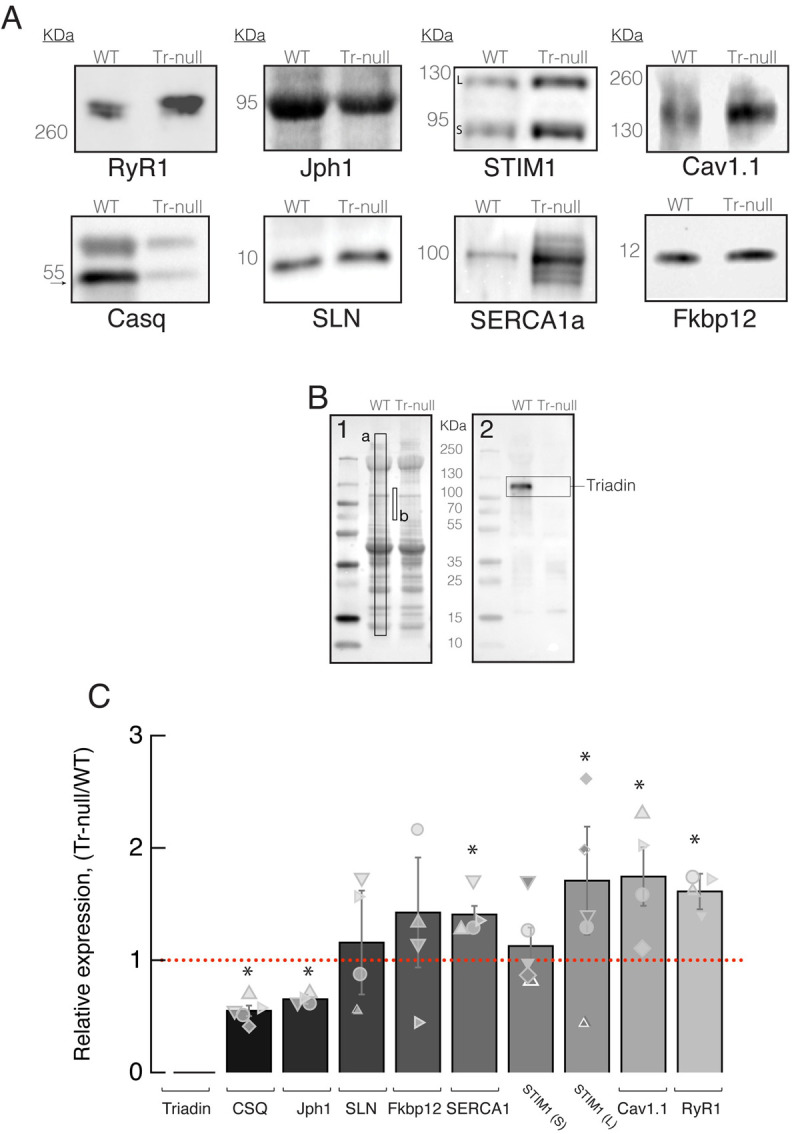
Effect of triadin ablation on the expression of couplon proteins. *A*, Western blot of proteins of interest from WT and Tr-null total homogenates. Band signal mass was normalized to signal in the corresponding lane of the gel in *B* (boxed region a corrected for baseline level in *b*). Details in Methods and [48]. *B*.1, Ponceau-stained proteins separated by PAGE. *B*.2, uncropped blot showing immunoreactivity of triadin antibody. *C*, distribution of results in Tr-null samples as a ratio of the WT average. Different symbols identify values obtained from individual mice. Error bars represent SEM. Asterisks mark changes statistically significant at the 0.05 (*) level. Statistical parameters are listed in Table 1.

Equal quantities of proteins (70 *μ*g for Ca_V_1.1 and RyR1 and 30 *μ*M for the rest of the proteins), were separated by SDS–polyacrylamide gel electrophoresis, and transferred to a nitrocellulose membrane (Bio-Rad, Des Plaines, IL). Membranes were blocked with 4.5% non-fat milk in PBS, and incubated overnight with primary antibody at 4°C. Thereafter they were incubated in horseradish peroxidase–conjugated anti-rabbit secondary antibody (Invitrogen, Carlsbad, CA) for 1 hour at room temperature. The blot signals were developed with chemiluminescent substrate (Millipore Sigma, Burlington, MA) and detected using the Syngene PXi system (Syngene USA Inc., Frederick, MD). The sources of antibodies were as follows: (MA3-927 (triadin), MA3-913 (Casq), MA3-920 (Ca_V_1.1), MA3-912 (SERCA1), MA3-925 (RyR1), MA5-32611 (FKBP12), and PA5-52639 (JPH1) (Invitrogen, Carlsbad, CA, USA), AB108994 (Stim1) (Abcam, Waltham, MA); ABT13 (Sarcolipin) (Millipore Sigma, Burlington, MA)). Quantitative analysis of Western blots was carried out with a custom application (written in the IDL platform) described and quantitatively validated by Tammineni *et al*. (2020) [48], which combines the information in the blot and the source gel. The content of the protein of interest was measured in the blot (Fig 1*A*) by the signal mass within a rectangle that enclosed the protein band. A normalization factor, quantifying the sample deposited in the lane, was computed on the gel (Fig 1*B*) as the average pixel signal in a large area of the corresponding lane (*a*) above a background (*b*).

**Table 1:** Levels of proteins expression.

	*Casq*		*Iph1*		*Ca*_*v*_1.1		*fkbp*12		*SERCA*1		*RyR*1		*Slpn*		*Stim*1	
	WT	Tr-null	WT	Tr-null	WT	Tr-null	WT	Tr-null	WT	Tr-null	WT	Tr-null	WT	Tr-null	WT	Tr-null
Expression level ± SEM	4.3 ± 0.5	2.3 ± 0.7	16.4 ± 0.5	10.7 ± 0.7	2.7 ± 0.5	4.7 ± 0.5	2.0 ± 0.7	3.1 ± 0.8	6.2 ± 0.5	8.7 ± 0.4	2.4 ± 0.2	1.5 ± 0.2	9.2 ± 2.6	11.5 ± 3.0	(L) 1.5 ± 0.6	(L) 4.1 ± 0.7
															(S)	(S)
															3.1 ± 0.7	3.5 ± 0.7
% ± SEM;	-46 ± 4.6		-35 ± 2.1		+74 ± 22		+42 ± 48		+40 ± 10		+62 ± 7.0		+15 ± 46		+71 (L) ± 48	
decrease (-), increase (+)															+12 (S) ± 16		
*p*, *N*	*p = 0.05, 5		*p = 0.003, 4		*p = 0.02, 4		*p* = 0.32, 4		*p = 0.005, 4		*p = 0.01, 4		*p* = 0.6, 5		*p(L) = 0.02, 5	

Quantitative analysis of proteins of interest. Expression levels of Tr-null proteins are represented as light intensity in units of convenience, and as a ratio of the WT average. % of expression represent the increase or decrease of protein quantities in Tr-null muscles with respect to WT.

### Statistics and presentation of replicates

The measurements that are compared in the present study are typically quantitative features of *N* individuals (mice) with *n*_i_ replications (myofibers) for individual mouse *i*. The total number of replications (myofibers, images, cases) used in a study is denoted as *m* = ∑*n*_*i*_. Statistical significance of the difference between variables was determined using hierarchical, *a*.*k*.*a*. nested analysis [49, 50]. Means are calculated conventionally, averaging over all measurements, for example:

$$\bar{V}=\overset{N}{\underset{i=1}{\sum }}n_{i}\overset{n_{i}}{\underset{j=1}{\sum }}V_{ij}/\overset{N}{\underset{i=1}{\sum }}n_{i}$$ (6)

Then, for evaluation of statistical significance of differences an effective number of cases is calculated, which corrects for the grouping or clustering of replicates from the same individual, thus avoiding pseudoreplication [50]. The effective number is less than the actual number of replicate measures (*m*, the denominator in Eq 6), which results in a greater SEM and greater value of the *p* of null difference. The nested analysis therefore prevents overestimation of significance while using the information available in all replicas.

The illustration of comparisons between measured variables is done with box plots, which show mean (dashed lines), median (bisecting line), bounds of box (75th to 25th percentiles), and whiskers representing 5% and 95% in the data set. All measures are plotted, identifying replicate measures from different individuals by different symbols. A special case is the comparison of spatial extent of terminal cisternae in junctions, which involves two levels of nesting, namely multiple images per myofiber and multiple myofibers per animal. This case required a modified statistical model (also exemplified in Sikkel *et al*. (2017) [49]), in a procedure referred to as three-level hierarchical analysis.

## Results

### Expression of couplon proteins and spatial extent of triadic region

The expression density of several couplon proteins was found to be different in the absence of triadin [11, 17, 35, 51]. Comparing Western blots of total *FDB* fractions (Fig 1) we found statistically significant increases of multiple couplon proteins in Tr-null myofibers, namely SERCA1: 40%, Stim1: 71% (long splice variant, “L”), Ca_V_1.1: 74%, and RyR1: 62% (Table 1 for summary). Two other proteins had lower densities; Jph1 was reduced by 35%, and Casq1 by 46%, a difference consistent with the 40% deficit reported by Boncompagni *et al*. (2012) [51], but less than the 73% loss reported by Oddoux *et al*. (2009) [11]. Finally, sarcolipin, which has modulatory effects on SERCA1 [54], remained statistically unchanged.

To test for possible changes in the proportion of fiber types, we evaluated the isoform composition of skeletal myosin heavy chain. As illustrated in S1 Fig and detailed in its legend, we found minor differences in type I, while the expression of types IIa and IIx were respectively 15% lower and 17% higher in the Tr-null. None of the differences were close to statistical significance.

Casq1 content is a determinant of TC volume [51, 52]. For an additional gauge of the structural impact of the triadin ablation, we evaluated the spatial extent of TC in z-stacks of confocal images of myofibers expressing CFP-Casq1 (S2*A* Fig). An average projection in the transversal direction was performed to generate a one-dimensional measure of the spatial extent of TC in the direction of the longitudinal fiber axis (*x* axis). This distance was calculated as the joint span of the t tubule plus the two TC of the projection on this axis of the marker fluorescence (S2*B* Fig). This calculation was done on one of the central images of the *z*-stack, after deblurring correction (Methods).

### Free Ca^2+^ concentrations in cytosol and SR

**Fig 2:**
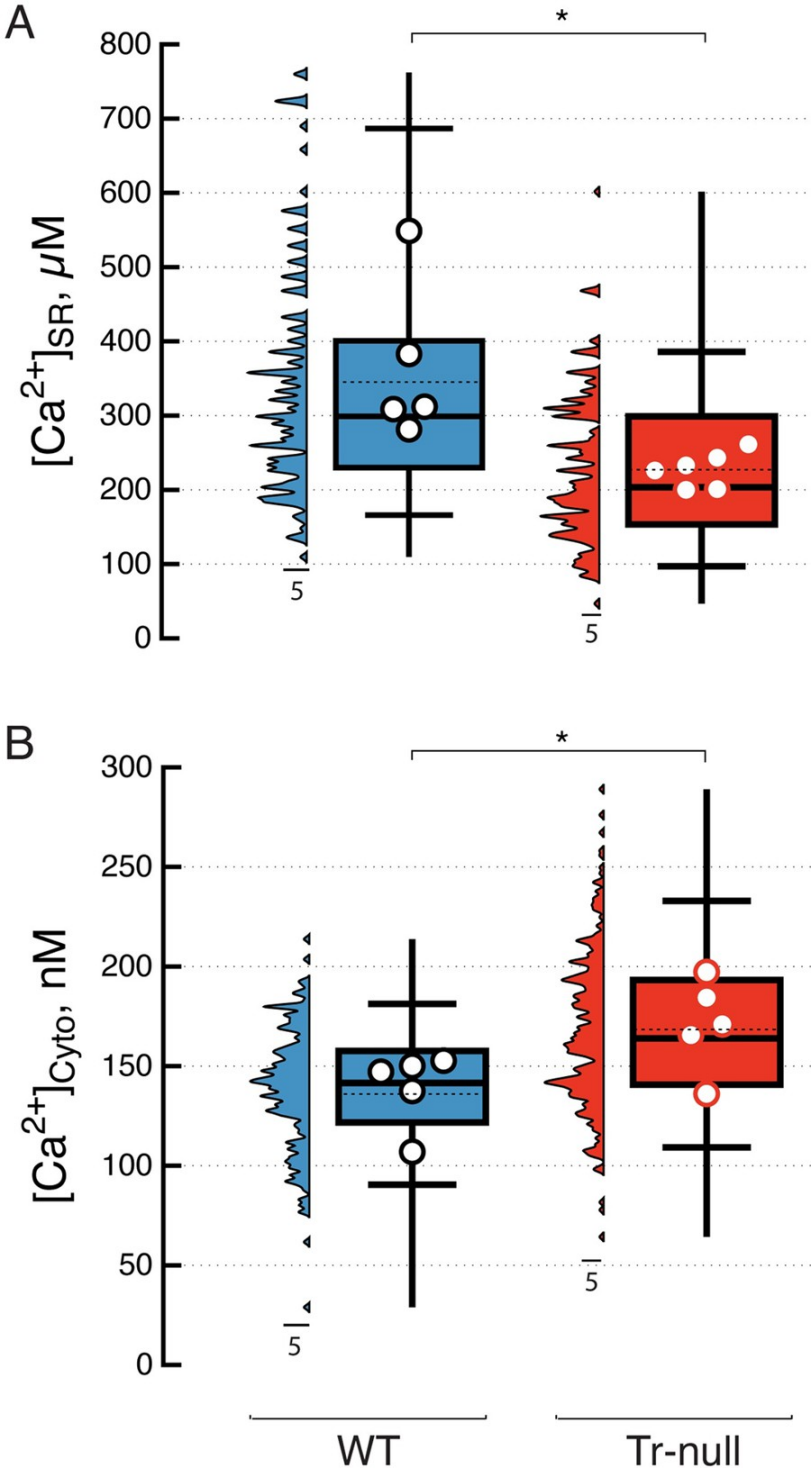
Resting calcium concentration in cytosol and sarcoplasmic reticulum. *A*, *B*, distributions of [Ca^2+^]_cyto_ and [Ca^2+^]_SR_, measured ratiometrically in WT and Tr-null myofibers. Multiple measurements are represented by the vertical histograms, while N symbols represent averages of measures in m myofibers of N mice and box plots summarize the distribution of individual measurements. *A*, average [Ca^2+^]_SR_: in Tr-null, 224 ± 9 μM (N = 6, m = 204); in WT, 343 ± 10 μM (N = 5, m = 162); *p* < 0.001 in three-level hierarchical analysis. *B*, averages of [Ca^2+^]_cyto_: in Tr-null, 167 ± 2 nM (N = 6, m = 291); in WT 139 ± 3 nM (N = 5, m = 195); *p* < 0.001.

Cytosolic calcium concentration [Ca^2+^]_cyto_, has been shown to increase in Tr-null adult myofibers and myotubes [25, 51], while SR calcium content is believed to be lower in Tr-null myofibers, based on the amplitude of Ca^2+^ transients induced by chemical RyR1 activation [11, 25, 51]. Here, resting [Ca^2+^]_SR_ was quantified directly, using SEER of the TC-targeted ratiometric biosensor D4cpV-Casq1 (Fig 2*A*). [Ca^2+^]_SR_ was about 35% lower in Tr-null than in WT myofibers (224 ± 9 μM; *vs*. 343 ± 10 μM). [Ca^2+^]_cyto_ (measured by SEER of Indo-1, Methods) was 20% higher in Tr-null fibers (167 ± 2; *vs*. 139 ± 3 nM, Fig 2*B*). Values are summarized in Table 2.

**Table 2:** Properties of Ca^2+^ release elicited by clamp depolarization.

	[Ca^2+^]_cyto_	[Ca^2+^]_SR_	${\dot{R}}_{P}$	*R*_*T*_	*R*_*P*_	*R*_*H*_
	(nM)	(μM)	(mM/s)	(μM)	(μM)	(μM)
**WT**	139	343	91.9	1655	835	820
*SEM; (N*,*m)*	*3; (5*, *191)*	*10; (5*,*162)*	*9*.*1; (9*,*17)*	*60; (9*,*17)*	*71; (9*,*17)*	*47; (9*,*17)*
**Tr-null**	167**	224**	92.3	1390*	796	593*
*SEM; (N*,*m)*	*2; (6*, *288)*	*9; (5*,*204)*	*10*.*3; (8*,*19)*	*65; (8*,*19)*	*54; (8*,*19)*	*41; (8*,*19)*

Ca^2+^ release elicited by a +30 mV, 100 ms depolarizing pulse. Listed are equal-weight averages and S.E.M. over N individual mouse averages of values found in m cells. Columns 2 and 3, summarize the resting cytosolic and intra luminal [Ca^2+^]. 4 to 7 list flux and quantities of Ca^2+^ released, defined as illustrated in Fig 4*B*. Asterisks mark variables that showed differences statistically significant at the 0.05 (*) or 0.001 (**) level.

### Properties of intramembrane mobile charge

**Fig 3:**
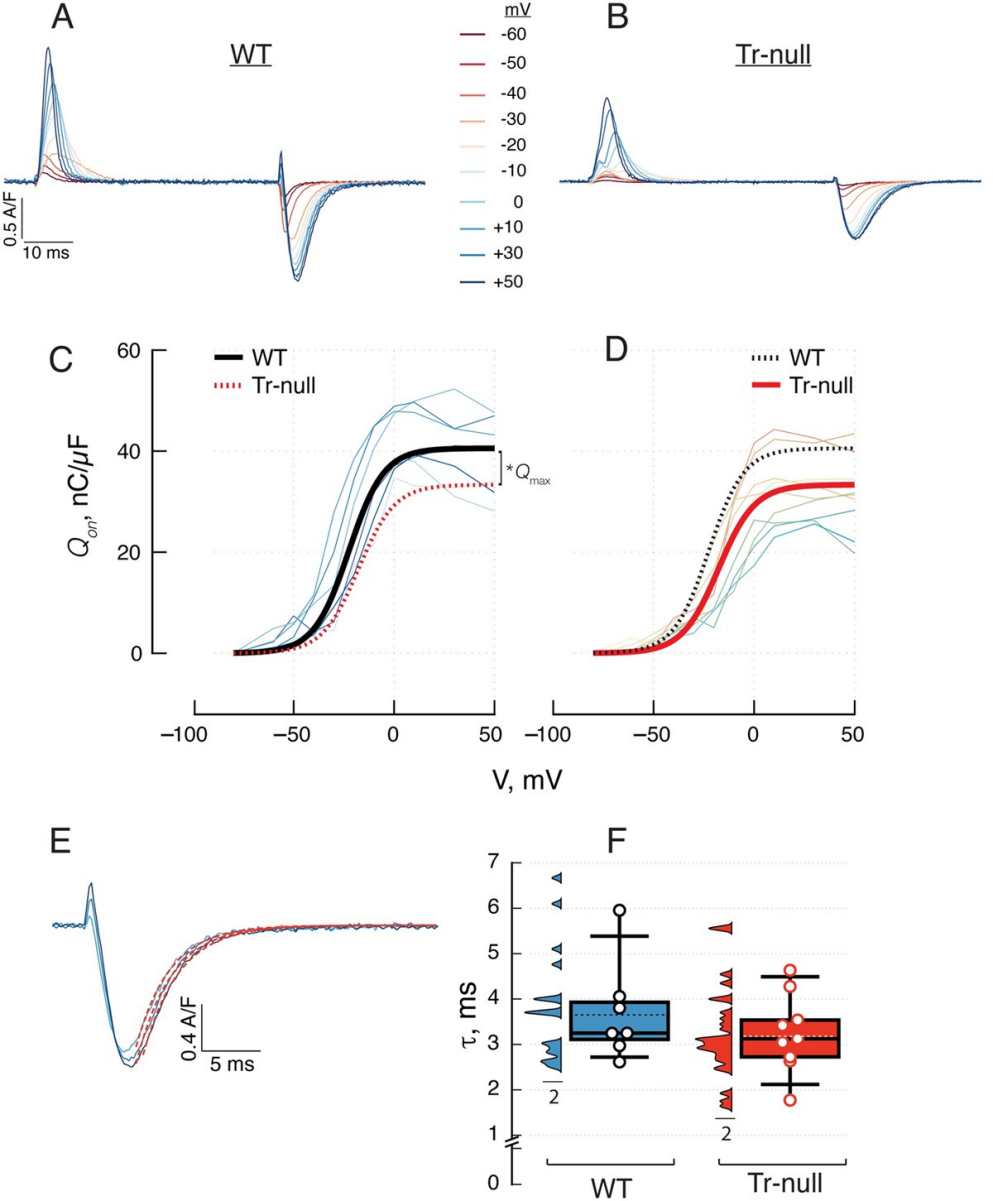
Voltage dependence of voltage sensor charge movement. *A*, *B*, representative charge movement currents obtained in myofibers held at -90 mV and depolarized for 50 ms to voltage V, ranging between -60 and +50 mV. *C*, *D*, charge displaced Q_on_, vs. V in WT and Tr-null fibers. Thin lines (measured values joined by straight segments) trace results in individual myofibers. These individual Q_on_(V) sets were fitted with a “Boltzmann” function. The thick smooth curves trace Boltzmann functions with parameters equal to the averages of parameters fitted to individual sets. Both average curves are plotted in *C* and *D* for ease of comparison. Details are presented in Table 3. *E*, kinetics; off sections of I_Q_(t) after depolarization pulses, with exponential fit to decay in red traces. *F*, distribution of fitted time constants. As in the preceding figures, symbols represent averages of measurements in individual mice, with replicates in m myofibers represented as vertical histograms.

The displacement of charge (*Q*) within the plasma membrane upon membrane depolarization was evaluated during the pulse (*Q*_*on*_) and after its end (*Q*_*off*_). The evaluation is illustrated in Fig 3. Pulses of 50 ms duration were applied from a holding potential of -90 mV to a final value, *V*, between -60 and +30 mV, in 10 mV increments. The “charge movement” current *I*_*Q*_(*t*,*V*) is presented in panels 3*A*, *B*. *Q*_*on*_(*V*), the time integral of the current during the pulse to voltage *V*, is plotted by thin lines for all WT fibers studied in panel 3*C* and all Tr-null fibers in 3*D*. The dependence of *Q*, *on* or *off*, on *V*, was fitted with a “Boltzmann” function (Eq 2) individually for different fibers (summary of values in Table 3). The smooth curves in thick trace in panels 3*C* and *D* plot the Boltzmann functions (Eq 2) generated with the averages of the parameters fitted for WT and Tr-null fibers respectively; for ease of comparison, the dashed curves in 3*C* and *D* replot the average-parameter Boltzmanns for Tr-null and WT respectively. As listed in Table 3, *Q*_*max*_ was about 20% smaller in the null, the central voltage $\bar{V}$ was displaced by 5 mV in the depolarizing direction, and the steepness factor increased by 20%. The difference in *Q*_*max*_ was statistically significant (*p =* 0.012); that in $\bar{V}$ was not (*p* = 0.06). Panel *E*, shows the *off* portion of intramembrane currents, in pulses to +10, +30 and +50 mV. The decay of these currents could be fitted with exponential functions of time (in red trace). The distribution of the exponential time constants is plotted in *F*. The decay in Tr-null was faster but the difference was not significant. The average τ was 3.2 ± 0.3, in Tr-null (*N* = 7, *m* = 10), *vs*. 3.8 ± 0.36 ms in WT (*N* = 7, *m* = 10); *p =* 0.25. *p* was calculated by two-level hierarchical analysis.

**Table 3:** Parameters of charge distribution and total calcium release.

	Charge distribution						ΔFF			*R*_*T*_		
	*Q*_*on*_(max)	*Q*_*off*_(max)	κ_*on*_	κ_*off*_	${\bar{V}}_{on}$	${\bar{V}}_{off}$	ΔFF (max)	κ	$\bar{V}$	*R*_*T*_(max)	κ	$\bar{V}$
	(nC/μF)	(nC/μF)	(mV)	(mV)	(mV)	(mV)	a.u.	(mV)	(mV)	(μM)	(mV)	(mV)
**WT**	41.3	-50.8	8.7	11	-22.4	-22.9	3.3	4.5	-24	1260	7.5	-21
*± SEM*	± 2.3	± 4.5	± 0.6	± 1.6	± 2.3	± 1.4	± 0.4	± 0.5	± 2.5	± 80	± 0.7	± 2.1
*(N*,*m)*	*(9*,*9)*	*(6*,*6)*	*(9*,*9)*	*(6*, *6)*	*(9*, *9)*	*(14*, *14)*	*(8*, *11)*	*(8*, *11)*	*(8*, *11)*	*(8*, *11)*	*(8*, *11)*	*(8*, *11)*
**Tr-null**	*32.3	*-42.6	9.1	11.9	-17.3	-16.1	2.5	5.3	-26	*1016	8.9	-23
*± SEM*	± 2.1	± 1.4	± 0.6	± 0.8	± 1.7	± 2.5	± 0.3	± 0.8	± 1.8	± 62	± 0.5	± 1.6
*(N*,*m)*	*(14*,*14)*	*(9*,*9)*	*(14*,*14)*	*(9*, *9)*	*(14*,*14)*	*(9*,*9)*	*(11*, *22)*	*(11*, *22)*	*(11*, *22)*	*(11*, *22)*	*(11*, *22)*	*(11*, *22)*

Charge distribution and total release parameters obtained after fitting the Boltzmann equation to charge mobilized or amount of calcium released by depolarizing pulses -as illustrated in Figs 3*C*–3*D* (columns 2 to 7) and 5*C-*5*D* & 5*G-*5*H* (columns 8 to 10 & 11 to 13), respectively-. Values are represented as the average ± SEM. *Changes significant at the 0.05 level.

In sum, the changes in intramembrane charge movement in the Tr-null were minor, except for the total mobile charge, which was reduced by 20%.

### Flux and quantity of Ca^2+^ released in Tr-null myofibers

**Fig 4:**
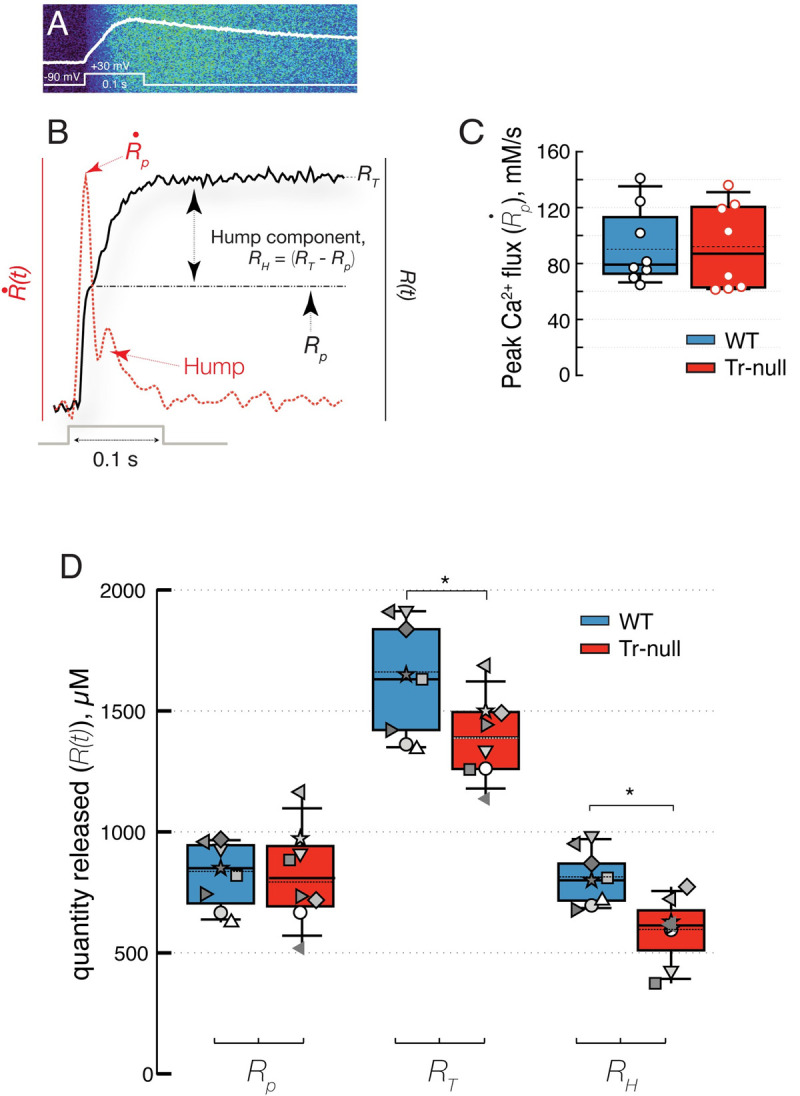
Calcium release elicited by maximal clamp depolarization. *A*, line-scan image F(x, t) of a WT myofiber of FDB muscle, upon application of the voltage pulse represented (+30 mV for 100 ms); the space average F(t) is overlaid. *B*, red: Ca^2+^ release flux $\dot{R}(t)$, derived from the [Ca^2+^]_cyto_(t) elicited by this pulse and others. The peak flux value is represented as ${\dot{R}}_{P}$. Black: quantity R(t) of Ca^2+^ released via voltage activation of RyR channels. Note definitions of the quantities R_T_, R_P_ and R_H_. A dip in the slope of R(t) (corresponding to the local minimum in $\dot{R}(t)$) marks the end of the peak stage and allows the calculation of R_P_. *C*, distribution of ${\overset{˙}{R}}_{P}$. The averages are nearly equal (~92 mM/s) in 8 Tr-null and 8 WT muscles. Details in Table 3: *D*, distribution of quantities R(t). Symbols represent values from line-scan images in separate myofibers; those from the same animal are represented by the same symbol. While the distribution of R_P_ was similar in Tr-null and WT, R_T_ was lower by ~16% in the null, with p = 0.011. As can be inferred from the difference in R_T_, the approximate constancy of R_P_ and the definition of R_H_, nearly all the difference in R_T_ was due to a smaller R_H_ component (*p* = 0.001). Fluxes and quantities of Ca^2+^ released at specific times were calculated from these records as illustrated in Fig 4*B–*4*D*; these include R_T_, the total quantity released up to the end of the pulse, R_P_, the calcium released during the peak stage, from the beginning up to the dip that precedes the hump, and R_H_, the calcium released during the hump stage, defined as R_T_−R_P_.

Ca^2+^ release flux was derived from calcium transients, [Ca^2+^]_cyto_(*t*), in turn calculated from the fluorescence imaged by confocal line-scanning of single muscle fibers, patched and loaded with Fluo-4. Fig 4 illustrates the measurement: panel *A* is the “linescan” image of fluorescence, *F*(*x*,*t*), upon application of a supramaximal depolarizing pulse (to +30 mV for 100 ms). The average over *x*, *F*(*t*), is overlaid in white. In 4*B* are Ca^2+^ release flux ($\overset{˙}{R}(t)$, red dashed trace) and the quantity of Ca^2+^ released (*R*(*t*), black trace). $\overset{˙}{R}(t)$ exhibits the normal features: a fast “peak” phase during which the flux reaches its absolute maximum ${\overset{˙}{R}}_{P}$, followed by a “hump”, which is the kinetic hallmark of calsequestrin’s contribution to Ca^2+^ release [34].

It was also possible to determine ${\overset{˙}{R}}_{H}$, the maximum flux reached during the hump phase; however, because of the small values and high variance of this variable, *R*_*H*_ was instead the one chosen for comparisons between WT and Tr-null muscles. ${\dot{R}}_{P}$, the maximum flux reached during the early peak stage, was not significantly different in Tr-null and WT muscles (Fig 4*C*), (Tr-null: 92.3 ± 10.3 mM/s; WT: 91.9 ± 9.1; *p* = 0.63). *R*_*T*_, the quantity released up to the end of the pulse (panel *D*), was significantly lower in the Tr-null muscles (Tr-null: 1390 ± 65 μM; WT: 1655 ± 60 μM; *p* = 0.011), a difference largely due to a smaller hump component *R*_*H*_ (Tr-null: 593 ± 41; WT: 820 ± 47 μM; *p* = 0.001). *R*_*P*_, the quantity released during the peak stage, was not statistically significantly reduced in the null.

### Voltage dependence of Ca^2+^ release

**Fig 5:**
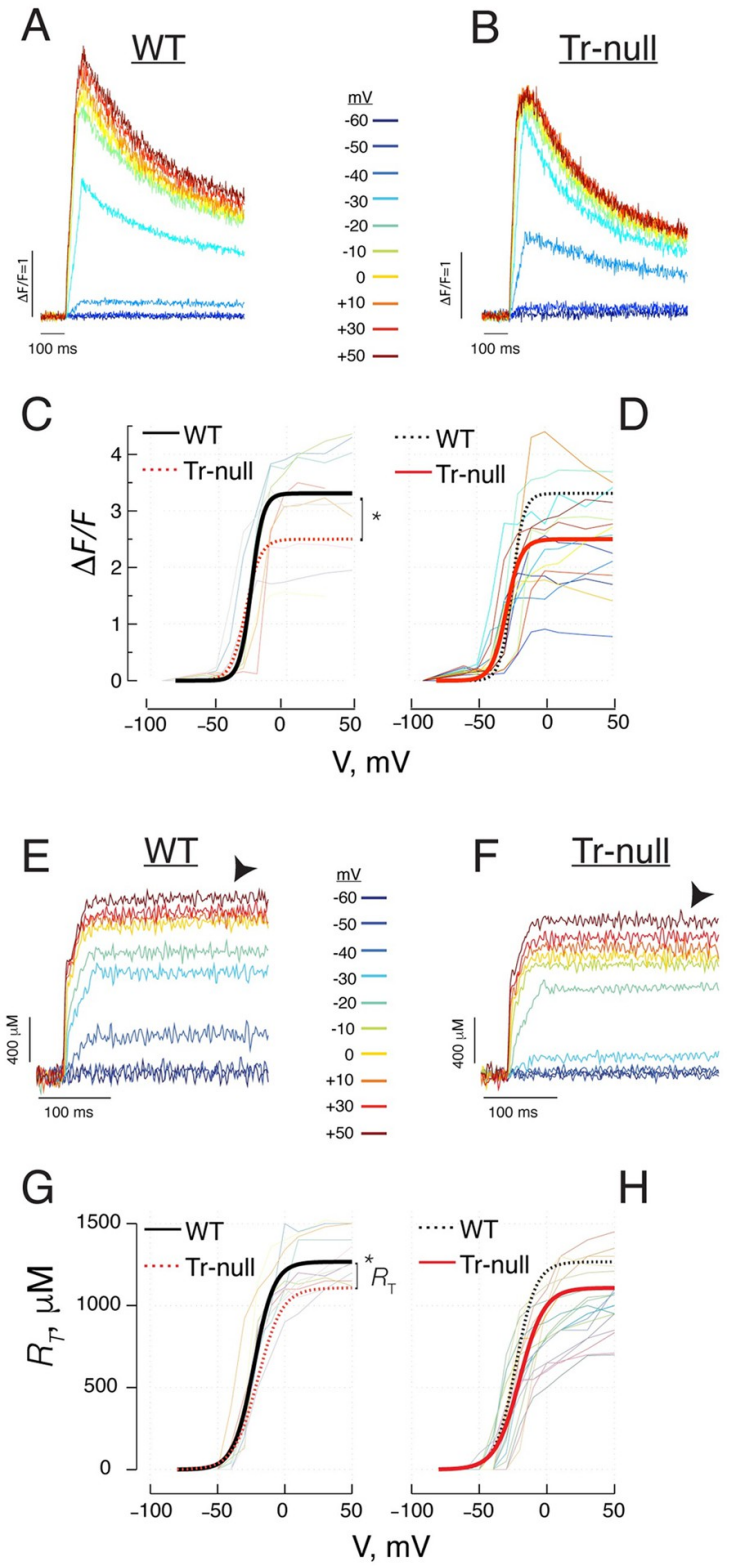
Voltage dependence of Ca^2+^ release. *A*, *B*, Fluo-4 fluorescence transients (*Δ*F/F_0_), upon 50 ms depolarizations to the voltages indicated. *C*, *D*, voltage dependence of fluorescence increases at end of pulses. Thin lines plot *Δ*F/F_0_(V) for every myofiber studied in WT (C) and Tr-null (D). Smooth curves trace the Boltzmann functions obtained with the averages of the best fit parameters of the same function fitted to the individual myofiber data. Average Boltzmann curves for both WT and Tr-nulls are repeated in *C* and *D* for ease of comparison. *E*, *F*, representative records of quantity R_T_ of Ca^2+^ released. *G*, *H*, voltage dependence of R_T_. Average Boltzmann curves for both WT and Tr-nulls are repeated in *G* and *H*. R_T_(max) was lower, by ~19%, in the Tr-nulls; the difference was statistically significant (*p* = 0.025). $\bar{V}$ was shifted to more depolarized values by 2 mV and κ was 16% greater in Tr-null myofibers. Statistical details are provided in Table 3.

Different variables of Ca^2+^ release can be used to characterize this dependence. We found that *R*_*T*_, the total quantity of calcium released by the end of the pulse, had the highest consistency among cells and individual mice. Representative results are illustrated with Fig 5. Panels 5*A* and 5*B*, show typical records of relative increase in fluorescence from which Ca^2+^ release was derived; in panels *E* and *F* we represented quantity *R*(*t*) upon depolarization pulses to a final voltage between -60 and +50 mV. The voltage dependence is represented in panels 5*C* and *D* by line plots *R*_*T*_(*V*) for all fibers studied (thin traces). A Boltzmann function (Eq 2) was fitted to the data in each fiber; the best fit fiber parameters were in turn averaged for comparison between WT and Tr-null groups. The group averages of the parameters were used to generate the smooth curves in Fig 5*C* and 5*D*. Comparing these panels with 3*C* and *D*, it can be seen that the difference in *R*_*T*_(*V*) between WT and Tr-null is consistent with that of *Q*(*V*). When comparing parameters between panels in Fig 5*G* and 5*H*, the average of the fitted maximum of *R*_*T*_ was significantly higher in WT fibers (Tr-null: 1016 ± 62 μM (*N* = 12, *m* = 22); WT: 1260 ± 80 μM (*N* = 9, *m* = 12), *p* = 0.02). The average half-maximum voltage ($\bar{V}$) was more positive in Tr-null fibers, but the difference was not significant (Tr-null: -21 ± 1.6 mV, (*N* = 11, *m* = 22); WT: -23 ± 2.1 mV (*N* = 8, *m* = 11), *p* = 0.64). While the steepness factor was greater in the null (8.79 ± 0.60 mV, *vs*. 7.46 ± 0.76 mV in the WT), the difference was not significant (*p* = 0.70).

### Recovery of Ca^2+^ flux after depletion

**Fig 6:**
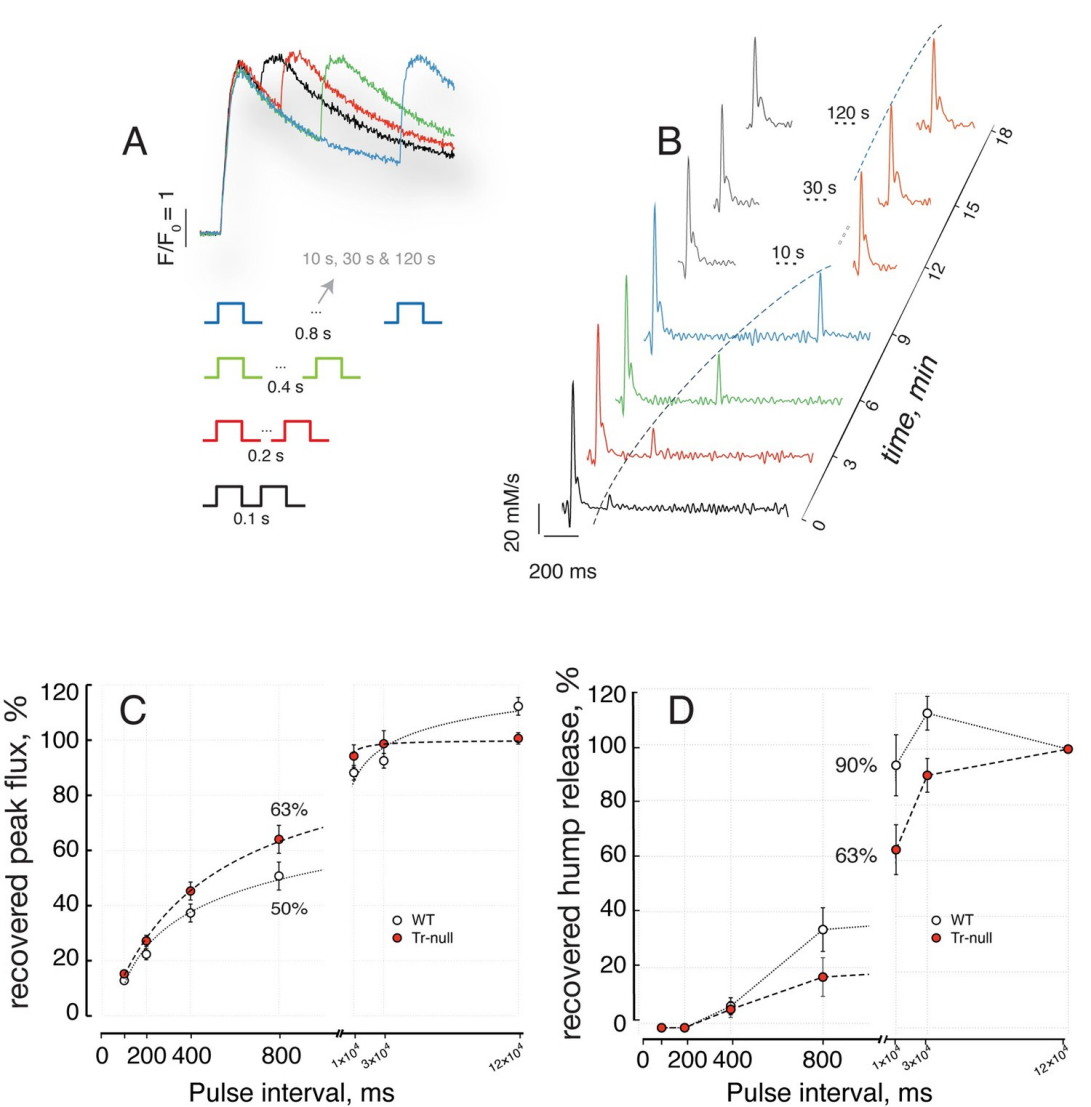
Recovery of Ca^2+^ release flux. *A*, fluorescence signals F(t) elicited by pairs of supramaximal pulses (+30 mV, 100 ms) separated by an interval ranging between 100 and 800 ms. The sets of paired pulses were separated by a resetting time of 3 minutes. *B*, Ca^2+^ release flux derived from the F(t) in *A*, plus flux elicited by 3 additional pairs of pulses at the greater intervals listed. *C*, peak flux ${\dot{R}}_{P}$ during the 2^nd^ pulse, represented as percent of ${\dot{R}}_{P}$ during the first one. *D*, quantity R_H_ in the hump stage of Ca^2+^ release elicited by the second pulse, represented as percent of R_H_ during the first one. The recovery of ${\dot{R}}_{P}$ is faster, while that of R_H_ is slower in Tr-null fibers.

The reduction in the hump phase of Ca^2+^ release points at the calsequestrin deficit as a cause of the functional changes in the Tr-null muscle. To characterize these changes further we determined the kinetics of recovery of peak flux and hump after a large depolarization, as this time course is sensitive to calcium buffering within the store. The experiments are illustrated in Fig 6. In panel *A* are fluorescence signals *F*(*t*), upon applying pairs of 100 ms pulses to +30 mV, at interpulse intervals ranging from 100 ms to 2 min. $\dot{R}(t)$, derived from each fluorescence signal in Fig 6*A*, is represented in 6*B*.

The flux elicited by the first pulse includes the usual peak stage followed by the hump. For short inter-pulse intervals, the flux in response to the second pulse in the pair has no hump; it consists largely of the peak stage, the amplitude of which increases with the interval between pulses; this recovery of the peak was shown in earlier work to reflect the progressive restoration of the [Ca^2+^] gradient that drives the flux from SR to cytosol [35].

As illustrated in panel 6*B*, the hump in the flux elicited by the 2^nd^ pulse only reappeared at 10 seconds and recovered almost completely after 30 s. Panels 6*C* and *D* summarize respectively rates of recovery of peak flux ${\overset{˙}{R}}_{P}$ and hump component *R*_*H*_ elicited by the second pulse. Recovery of ${\dot{R}}_{P}$ occurs more rapidly in Tr-null myofibers, reaching 63% of the un-conditioned value after 800 ms, *vs*. 50% for the WT. The recovery is almost 100% after 10 s. Changes in hump recovery were opposite to those of peak flux; *R*_*H*_ was restored at a slower rate in Tr-null fibers, to 63% after a 10 s interval, *vs*. 90% in WT. The divergence in hump recovery is especially significant because it occurred in spite of the faster recovery of free [Ca^2+^]_SR_ in the Tr-null, as evidenced by the rate of restoration of peak flux (summarized in Table 4).

**Table 4:** Ca^2+^ release elicited by two successive pulses.

	${\dot{R}}_{P}\;(mM/s)$		*R_T_* (*μM*)		*time constants* (*τ*)	
	1^*st*^ *Pulse*	2^*nd*^ *Pulse*	*R*_*T*2_	100*RT2RT1	[*Ca*^*2+*^]_*cyto*_ *(ms)*	*R*_*Net*_
						*(s)*
***WT***	68	34.5	1470	32.4	523.8	3.8
*SEM; (N*,*m)*	*5*.*7; (5*, *8)*	*3*.*4; (5*, *8)*	*61; (5*, *7)*	*3*.*8; (5*, *7)*	*32*.*8; (8*,*15)*	*0*.*19; (8*,*15)*
***Tr-null***	62.2	39.8	1226*	48.1*	435.6*	2.8**
*SEM; (N*,*m)*	*6*.*1; (6*, *8)*	*5; (6*, *8)*	*57; (6*, *8)*	*2*.*8; (6*, *8)*	*28*.*5; (9*,*16)*	*0*.*04; (9*,*16)*

**Fig 7:**
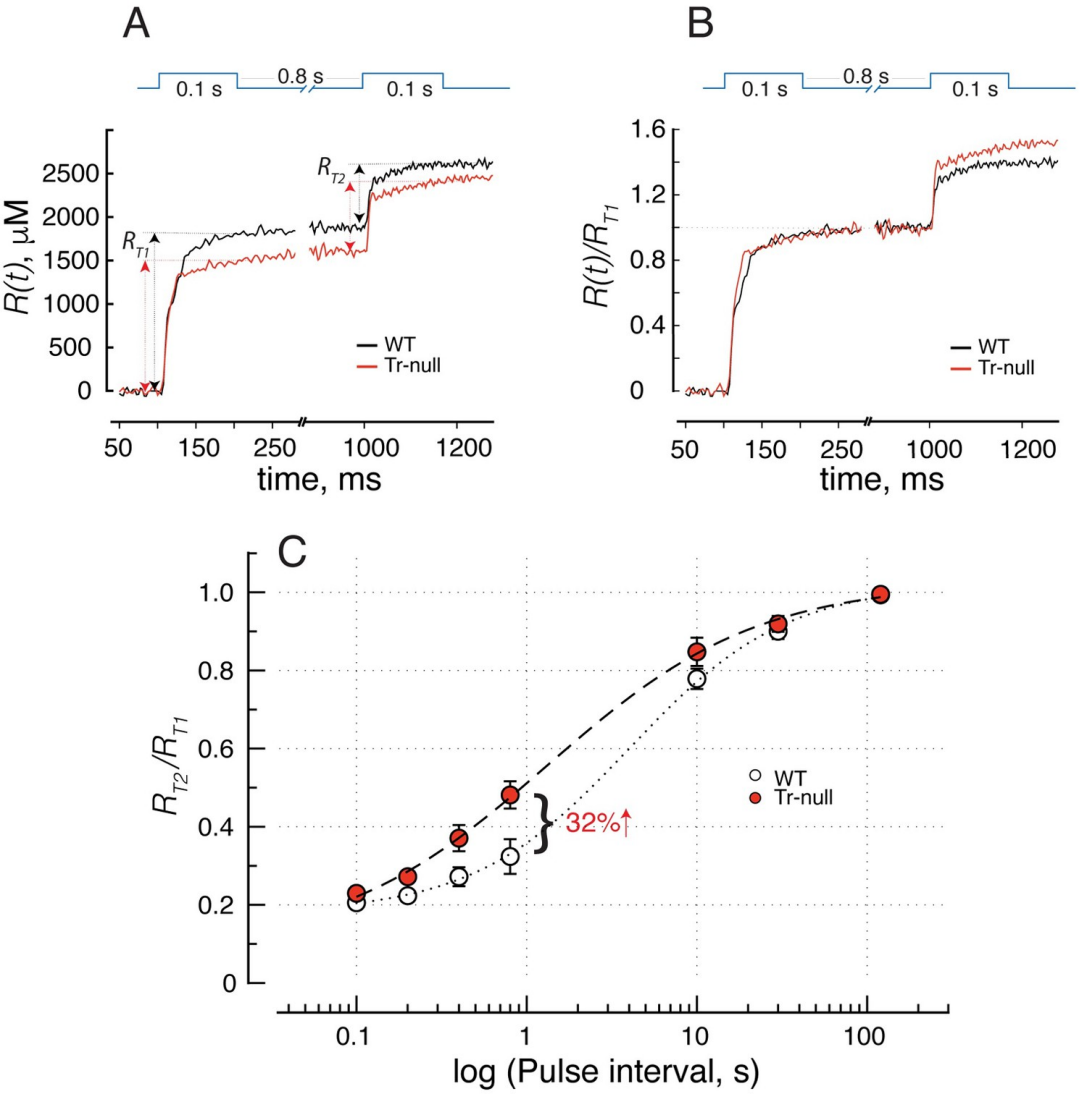
Recovery of the quantity of Ca^2+^ released. *A*, R(t) elicited by a pair of pulses 800 ms apart. Two-head arrows represent maximum quantities (R_T1_ and R_T2_) released with the respective pulses. *B*, data from A as fractions of quantity R_T1_. *C*, R_T2_ as fraction of R_T1_. Normalized thus, the quantity of Ca^2+^ released is ~32% greater in Tr-null at the 800 ms interval. The superposition of WT and null traces marks clearly the end of the peak stage and allows an accurate measurement of the quantity of Ca^2+^ contributed by the hump, as the difference between the total, R_T1_, and the level at the start of the hump. The dependence of R_T2_ on pulse interval is represented and summarized in panel *C*. This difference is in the same direction as that noted for peak flux; both suggest faster recovery of SR luminal free [Ca^2+^] in the absence of triadin.

**Fig 8:**
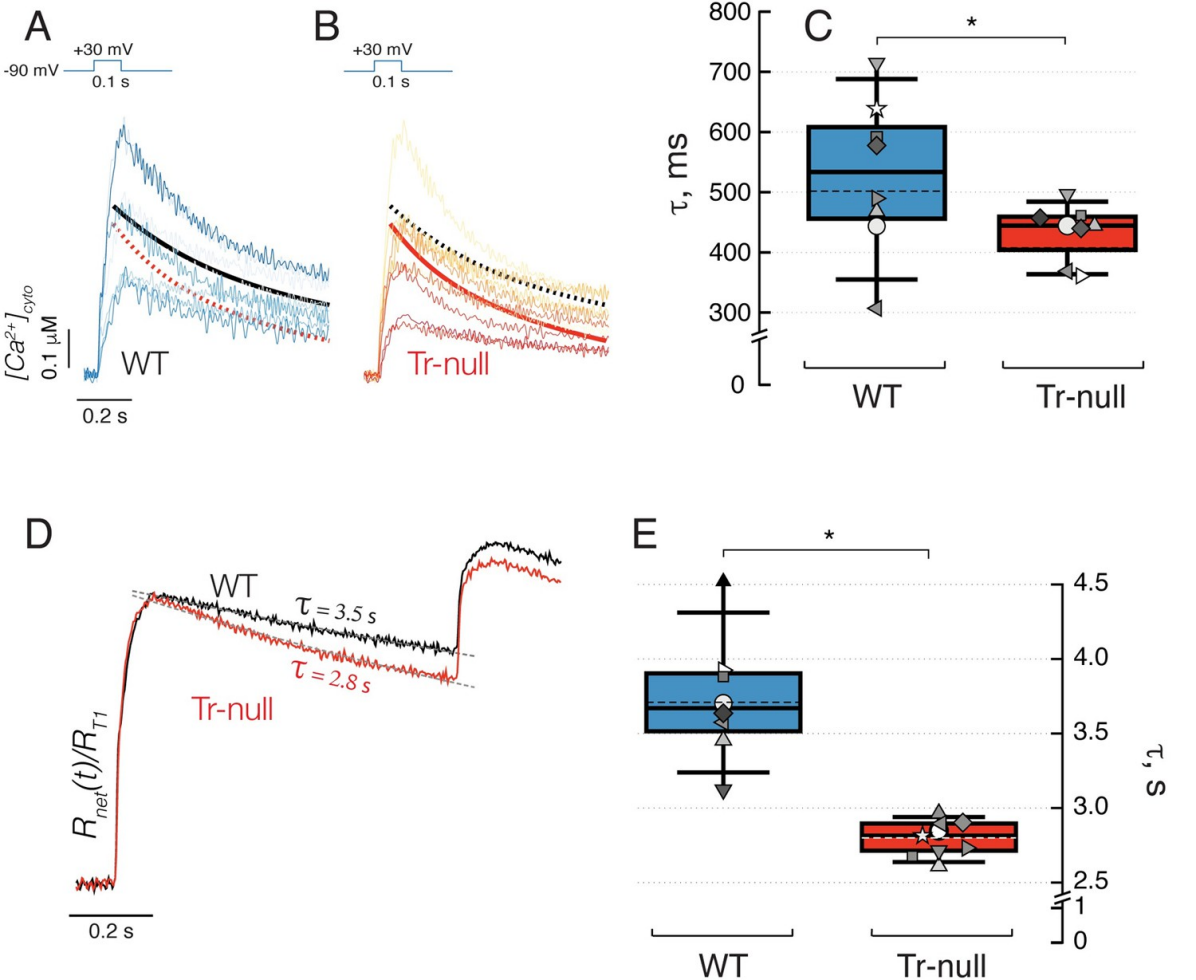
Kinetics of decay of cytosolic Ca^2+^. *A*, *B*, increase in [Ca^2+^]_cyto_(t) elicited by a supramaximal pulse. Traces from 8 WT animals are shown in *A* and 9 Tr-null in *B*; continuous smooth curves are exponential decay functions obtained with the average parameters of exponentials fitted individually to the decay of each myofiber (in *A*, solid black trace: WT and in *B*, solid red trace: Tr-null). WT and Tr-null curves are repeated in *A* and *B*. *C*, distribution of individual time constants in *A*, *B*; averages ± SEM (Tr-null: 434 ± 16. WT: 527 ± 45 ms. p = 0.04). *D*, Net quantity of Ca^2+^ released upon paired voltage-clamp pulses 800 ms apart (calculated from all records in *A* and *B*) shown normalized to the total quantity released by the first pulse, R_T1_. Consistent with the analysis illustrated with Fig 7, the 2^nd^ pulse releases more Ca^2+^ (as a fraction of that released in the first pulse) in the Tr-null. *E*, distribution of individual time constants of decay. Their average is ~36% greater in the WT (3.79 ± 0.19 s in WT vs. 2.79 ± 0.04 in the null; *p* < 0.001). Similar numbers are found by analyzing the decay of the average time courses, as shown in panel *D*. The large difference between time constants of [Ca^2+^]_cyto_ and R_net_ is justified in Discussion.

Parameters obtained after applying two pulses (to +30 mV, 100 ms duration) separated by an interpulse time of 800 ms. Columns 2 and 3 list averages of peak flux elicited by the corresponding pulse, as shown in Fig 6*B*. Columns 4 and 5 list the amount released by the second pulse and the value as percentage of the total in the first pulse (Fig 7*A* & 7*C*). Finally, columns 6 and 7 list the time constants of decay of the calcium transient (Fig 8*C*), and decay of the net quantity of calcium released (Fig 8*E*), a decay that reflects recovery of SR content at a time when release channels are closed. Asterisks mark variables that showed differences statistically significant at the 0.05 (*) or 0.001 (**) level.

The total quantity *R*_*T*2_ of Ca^2+^ released by the second pulse in a pair also recovered differently in Tr-null cells. To understand the implications of this difference it is necessary to keep in mind that the pulses are of supramaximal voltage (fully activating the release permeability) and of duration sufficient to elicit evacuation of a large fraction of the total SR Ca^2+^ available for release by depolarization. Thus, they constitute a measure, albeit approximate, of Ca^2+^ content available to be released. The time courses of quantity of Ca^2+^ released by a pair separated by 800 ms are illustrated in Fig 7*A*. The total quantities released by the individual pulses, *R*_*T*1_ and *R*_*T*2_, are clearly separable in the traces. *R*_*T*2_ was generally lower in Tr-null fibers at the intervals evaluated, which is consistent with a lower SR calcium content at all times. By contrast, as a fraction of *R*_*T*1_—the total quantity of Ca^2+^ released by the first pulse—the release of Ca^2+^ by the second pulse was greater in the Tr-null. This is illustrated in panels 7*B*, showing the normalized traces *R*(*t*)/*R*_*T*1_. The excess release in the Tr-null as a fraction of the total releasable load is maximal at intervals near 1 s, as shown in panel 7*C*. Because the quantity released by the 2^nd^ pulse measures most of the releasable content of the SR at the time, it follows that content recovery after a pulse is faster in the Tr-null muscle.

### SR uptake of released Ca^2+^

The changes in various aspects of Ca^2+^ release observed in Tr-null muscles suggest a faster recovery of free [Ca^2+^]_SR_. An unchanged flux of uptake by SERCA1 accumulating Ca^2+^ in a lumen with less Casq1 would result in a faster recovery of both free and total SR Ca^2+^ levels. An increased activity of the Ca^2+^ pump, expected from the increased content of SERCA1 in microsomes of Tr-null mice ([17] and Fig 1), would contribute further to the observed changes. Pump activity was evaluated through the kinetics of decay of Ca^2+^ transients after a supramaximal pulse (see Table 4). The comparative study, on the same records used for characterizing recovery of Ca^2+^ release, is illustrated in Fig 8. Panel *A* shows individual [Ca^2+^]_cyto_(t) records, superimposed after normalizing to the value at first peak, with single exponentials fitted to the decay phase. On average, the decay was faster in Tr-null, as represented in panel *B* (statistic parameters in Table 4). The difference in exponential time constants *τ*_*cyto*_ was statistically significant (436 ± 28 ms for Tr-null *vs*. 524 ± 33 ms for WT; *p* = 0.04).

The “removal” method used here for derivation of release flux [44, 53–55], requires describing quantitatively the fluxes of Ca^2+^ release, Ca^2+^ uptake by organelles and its binding to various targets. This quantitative framework is suitable for comparisons of SERCA pump flux (the estimates are less reliable in absolute terms, because they depend on variables not fully defined in these experiments, as discussed in [45]).

In the simplified form used here, the model that describes the fluxes is closed to the exterior (it neglects flux across the plasma or TT membrane) and only has two compartments. One is the cytosol, where Ca^2+^ buffers and binding sites are subsumed into one, “BAPTA”, as the internal solution has sufficient BAPTA that other ligands can be neglected for short-term analysis. The other is a storage compartment, named the SR but comprising all Ca^2+^ removing organelles (again, the dominance of the SR as storage device is well established). Within this simple framework, *R*(*t*), the Ca^2+^ released by the voltage pulse, differs from *R*_*net*_(*t*), the net released Ca^2+^, because the SERCA pump removes Ca^2+^ from the cytosol at all times, including that when release is proceeding. The formal calculation of *R*_*net*_ is done as described in Methods. Applied to the records in Fig 8*A* and 8*B*, the calculation yields the records shown normalized in Fig 8*D*.

After the end of the pulses, [Ca^2+^]_cyto_ is seen to decay monotonically. *R*_*net*_ also decays, mirroring recovery of SR content. Within the simple theory used here, this decay is largely determined by SERCA transport. The rate of decay is faster in the Tr-null. As documented with panel *E* and summarized in Table 4, the difference is highly significant. The change of average time constants $\tau_{R_{net}}$ (Tr-null: 2.8 ± 0.04, *N* = 9; WT: 3.8 ± 0.19 s, *N* = 8; *p* < 0.001) corresponds to a 26% increase in uptake rate in null muscle, in rough agreement with the change in SERCA1 content of Tr-null total fractions, Fig 1*C*, and with earlier results of Boncompagni *et al*. (2012) [51]. The observations on Tr-null cells suggest that their faster recovery of release flux after conditioning by an SR-depleting pulse is due to an increased activity of SERCA1, working to replenish an SR that contains less (Casq1) Ca^2+^ buffer.

## Discussion

The deletion of triadin results in functional deficits that were evaluated in several studies. Upon the description of the hump in Ca^2+^ release flux and the identification of Ca^2+^ bound to Casq1 as its source, we realized that the changes in this kinetic component offered the possibility to quantify the deficit in Ca^2+^ release attributable to disruption of this source in Tr-null muscle. This quantification is the primary goal of the present study.

Because the Tr-null mouse line used was different than those in previous studies, we first verified the ablation of triadin and quantified the expression of couplon and Ca^2+^ transport proteins, already known to be altered in Tr-null mice. We found similarities in protein expression in FDB, EDL, and hind leg muscles, as previously reported by Shen et al. (2007) [25], Oddoux *et al*. (2009) [11], and Boncompagni *et al*. (2012) [51]. Generally, they were overexpressed in the Tr-null; however, the excess proteins found in the null muscles varied in different models. For instance, we found the overexpression of RyR1 and Ca_V_1.1 to be 62% and 74% in Tr-null FDB, whereas, with EDL, both, Shen *et al*. (2007) [25] reported 20% and 15%, and Oddoux *et al*. (2009) [11] 25% and 70%, respectively. Working with hind leg muscles, Boncompagni *et al*. (2012) [51] reported 40% and 20%, excess in these two proteins. The SERCA excess, found in this work (40%) was similar to that reported by Boncompagni *et al*. (2012) [51] (30%), but greater than that reported by Oddoux *et al*. (2009) [11] (4%). Differences found in protein endowment between other studies and this one may be ascribed to the use of different muscles, or to slightly different compensation for triadin ablation in the different models.

It has been previously shown by electron microscopy that Casq1 is partially misplaced from the junctional SR in Tr-null fibers [35]; we also explored the distribution of Casq1 within the sarcomere to monitor, possible changes associated with loss of its putative anchor within the limitations of our optical level of resolution.

### The number of functional voltage sensors was reduced in Tr-null cells

In spite of the increase in expression of Ca_V_1.1 (Fig 1), we found a statistically significant reduction, by ~20%, in the density of functional voltage sensors in t tubule membranes, as measured by the maximum mobile intramembrane charge per unit of membrane capacitance (Fig 3). This reduction was accompanied by a shift of charge displacement to higher voltages, by about 5 mV. The shift was not significant statistically; if real, it should be minor, as suggested by the even smaller and also not statistically significant positive shift detected in the voltage dependence of activation of Ca^2+^ release (Fig 5).

### Calsequestrin expression is reduced in Tr-null cells

The present measurements, by Western blotting on total proteins homogenates from FDB muscle, found a 46% decrease in Casq1 levels in Tr-null mice. This decrease is greater than that found in Tr-null mice EDL [25], but it is in agreement with a previously reported result [51].

Oddoux *et al*. (2009) [11] and Boncompagni *et al*. (2012) [51] also found in EM images of Tr-null myofibers a reduction in TC diameter (the dimension perpendicular to the long axis of TC as seen in longitudinal myofiber sections). The change is consistent with a reduction in Casq1 content, given the consensus that this content is a determinant of SR volume and structure [35, 56]. We assessed any corresponding changes in the live cells by the thickness of the images of fluorescence of a tagged Casq1 in triad junctions. Although at this level of resolution we were unable to observe any displacement of Casq1 towards the longitudinal SR, we found an average reduction of ~19% in the Tr-null width, consistent with the published EM studies [51, 57]. The changes in Casq1 endowment, confirmed by our study, oriented much of the work presented here towards the exploration of possible effects of loss of the storage protein.

### Tr-null muscle had significant changes in Ca^2+^ distribution at rest

#### The cytosolic Ca^2+^ concentration was elevated in Tr-null myofibers

In the present study, [Ca^2+^]_cyto_ was found to be significantly higher in the Tr-null. The increase, 17%, is in rough agreement with the 28% increase found by Shen *et al*. (2007) [25] in mouse *tibialis anterior*; the excess [Ca^2+^]_cyto_ found in these muscles was substantially lower than that found in Tr-null primary myotubes (~50%; [51]).

To consider possible mechanisms for the increase, it is useful to keep in mind that the proximate cause must be a change in the properties of the plasma or t tubular membrane, as required by the Cell Boundary theorem [58]. While other mechanisms cannot be ruled out, an increase in Ca^2+^ permeability of the plasma membrane is a known consequence of a decay in SR free Ca^2+^ concentration [59].

**The SR Ca**^**2+**^ **concentration was lower than normal in Tr-null myofibers.** We measured a 35% lower [Ca^2+^]_SR_ in Tr-null than WT muscles; this difference is consistent with the lower Ca^2+^ releasable by depolarization found in the present study and with the difference in Ca^2+^ releasable by caffeine or depolarization by high potassium, found in myotubes [11, 25, 51]. A reduction in [Ca^2+^]_SR_ by 1/3 of the resting value was found to activate SOCE in skeletal muscle [60]; therefore, an activation of SOCE probably contributes to the higher [Ca^2+^]_cyto_ in Tr-null muscle.

To complete the interpretation, the observed difference in [Ca^2+^]_SR_ should be justified in the context of triadin ablation. The following are plausible reasons for the lower [Ca^2+^]_SR_: i) the absence of triadin could remove a direct inhibitory effect on RyR1, for which there is evidence [25, 61, 62]. ii) The deficient functional FKBP12/RyR1 interaction, demonstrated in Tr-null muscle [18], should reduce the stability that this subunit confers to the closed state of RyR1, with the consequent leak of SR calcium [18]. iii) Even though the effects of Casq1 on RyR activity are still debated, a variety of studies of muscle with ablation of Casq1 [25, 31, 63] or triadin [18], and reconstituted interactions in bilayers and vesicular fractions, support an inhibitory effect of the couplon proteins downstream from the RyR. It could be a basal inhibition (evinced by the studies in bilayers, e.g. [30]), or a closing effect activated by depletion, with Casq1 acting as a calcium sensor [31, 32]. Casq1 is also a negative regulator of the interaction between STIM1 and Orai1 [64, 65]. Removal of this effect could contribute to activation of SOCE and the increase in [Ca^2+^]_cyto_ observed in Tr-null muscle.

In addition to STIM1-Orai1, other pathways could be activated in Tr-null muscles, such as transient receptor potential channels (TRPC), as suggested by the blocking of both pathways of Ca^2+^ entry by 2-APB and BTP-2 [18].

The tentative conclusion in this section is that the increase in [Ca^2+^]_cyto_ results from changes at the plasma membrane consisting mostly in activation of SOCE in response to a reduction in [Ca^2+^]_SR_ due to loss via RyRs. In turn, the increased propensity to open of the RyRs may be a release from either a basal inhibitory effect of triadin or an inhibitory effect of Casq1, mediated by triadin.

#### The permeability of the SR Ca^2+^ releasing membrane was greater in Tr-null muscle

Neither the initial peak of release flux nor its time integral *R*_*P*_ were changed significantly in the Tr-null. This was unexpected: Ca^2+^ flux is proportional to the free [Ca^2+^] gradient between SR lumen and cytosol [32], a gradient reduced by about 35% in these Tr-null muscles. The approximate conservation of peak flux in spite of a lower driving [Ca^2+^] gradient amounts to an increase in peak permeability of the SR releasing membrane.

#### The deficit in Ca^2+^ release in Tr-null muscle was less than predicted

In Tr-null muscle, *R*_*T max*_ (total Ca^2+^ released by a 100 ms depolarization to a supramaximal voltage) was 265 μM or 19% lower than in WT. The difference was fully accounted for by the component *R*_*H*_ (quantity released in the hump stage), which was lower by 227 μM, or 27% of the WT value. The Ca^2+^ released in this phase detaches from Casq1 (as demonstrated by Manno *et al*. (2017) [35]). The deficit in *R*_*H*_, however, was less than that in Casq1 expression (46%).

The discrepancy between Ca^2+^ release deficit and reduction in Casq1 endowment, in addition to the non-functional Casq1 misplaced from the junctional SR, was compounded by the ~30% lower level measured for [Ca^2+^]_SR_. In the hypothesis of proportionality between releasable SR Ca^2+^ content and free [Ca^2+^]_SR_, this change alone should reduce the hump by 30%; however, the Ca^2+^ buffering power of the SR is known to decrease with free concentration of the ion [27, 66]; therefore, if Casq1, the source of the hump, is reduced to 0.6 of its WT value, and its buffering power is reduced in approximately the same proportion, its ability to release Ca^2+^ should be reduced by more than one half. That we did not find a corresponding reduction in releasable calcium is evidence of powerful compensation mechanisms operative in these animals to preserve the ability of the SR to release calcium. Even though the nature and kinetics of these mechanisms remain unknown, they could be assigned to the presence of other SR Ca^2+^-binding proteins, which contribute to reversible Ca^2+^ storage [67], and might increase their buffering power as [Ca^2+^]_SR_ is lowered (as would be expected for ligands of high Ca^2+^ affinity).

That the number of functional voltage sensors was also reduced in Tr-null muscle (Fig 3) further compounds the difficulty in reconciling the modest decrease in Ca^2+^ release with the other functional and structural changes observed. The presence of four Ca_V_1.1 units per RyR1 channel leaves room for a possible functional reserve; in this view, channels would open even if acted upon by an incomplete set, with fewer than 4 functional, charge-carrying Ca_V_ units. A recent theory of physical interactions between RyR1 and Ca_V_1.1 [68], provides a framework for the operation of release channels with an incomplete set of sensors. The possibility is interesting in the present case because Tr-null myofibers show an important increase in the expression of RyR1 and multiple indications that the absence of triadin relieves the channels from steady inhibition. The combined observations provide some support for the following speculation: a lower number of functional voltage sensors, strategically distributed among a greater number of release channels more prone to opening, achieves greater activation, thus justifying the observed increase in peak permeability of the SR Ca^2+^ releasing membrane.

### The contribution of calsequestrin to calcium storage and release

The discrepancy between the modest observed reduction in Ca^2+^ release and that predicted from the joint reduction in Casq1 content, free [Ca^2+^]_SR_ and functional voltage sensors, calls into question the assumptions used in the literature to calculate the fraction of releasable Ca^2+^ contributed by Casq1. We previously estimated it at 75%, equal to the reduction in releasable Ca^2+^ in Casq1-null mice relative to wild-type [27]. This estimate required the assumption that the Casq1 nulls have no change in other proteins (for instance a compensatory increase), in turn based on the relative invariance of known Ca^2+^-binding proteins, reported since the initial work with Casq1-null mice [57]. We now find evidence of substantial compensation mechanisms that increase Ca^2+^ release in Tr-null mice. If similar mechanisms operated in the Casq1-null, the contribution of Casq1 to releasable Ca^2+^ in the wild-type should be revised higher. A greater contribution of Casq1 would in turn enlarge the discrepancy found here between expected and observed releasable Ca^2+^, hence also strengthening the evidence of compensatory changes in the muscles with constitutive triadin knockout.

The time course of recovery of Ca^2+^ release following a depleting stimulus revealed an additional component of the alterations caused by ablation of triadin. In previous work we showed that the recovery of the peak flux (and quantity of Ca^2+^ released during the initial “peak” stage) parallels the recovery of [Ca^2+^]_SR_ measured directly [35], which indicates that the initial stages of release are fed by Ca^2+^ that is already in solution, or bound in rapid equilibrium with free Ca^2+^. We also showed that recovery of the hump is much slower [35], and its time course is similar to that of polymerization of Casq1, as inferred from FRAP (fluorescence recovery after photobleaching) of the tagged protein. The present result, faster recovery of Ca^2+^ flux and quantity released at the peak in Tr-null muscles, is consistent with their reduced Casq1 content, because the rise of free [Ca^2+^]_SR_ should be faster if Ca^2+^ were pumped into the SR at the same rate and had to satisfy a diminished buffer capacity in the SR lumen. That the exponential rate of decay of [Ca^2+^]_cyto_ was somewhat increased in null muscles points at enhanced SR Ca^2+^ uptake rate as another contributor to the increased rate of Ca^2+^ release recovery, and is in agreement with the increased expression of SERCA1 (present observations and [35]).

We also observed a slowing of recovery of the hump component of Ca^2+^ release, or what was left of it. This change reflects disruption of the Casq1 repolymerization process required to regain the storage capacity needed to contribute the hump of Ca^2+^ release [35]. The disruption could be due to the reduction of Casq1 concentration and/or that of triadin, as the protein is believed to provide nucleation centers that propel the polymerization process [35].

### Ca^2+^ buffering power of the cytosol

The analysis of decay of [Ca^2+^]_cyto_ provides a way to calculate the effective Ca^2+^ buffering power of the cytosol. Indeed, the time constants of decay of both [Ca^2+^]_cyto_ and *R*_*net*_ are lower in Tr-null muscles, reflecting faster Ca^2+^ uptake; however, the time constants, of hundreds of ms for the cytosolic concentration change and a few seconds for *R*_*net*_ (which reflects replenishment of SR content) differ by nearly an order of magnitude. In the framework of the analysis, the different rates reflect different buffering in the two compartments.

Indeed, the rate of change of free concentration in the cytosol can be written as

d[Ca2+]cytodt=−uptakefluxBcyto (7)

where *B*_*cyto*_ represents the buffer power of the cytosol, defined as

Bcyto≡d[Ca]cytod[Ca2+]cyto (8)

namely, the derivative of total cytosolic calcium concentration with respect to free calcium concentration. After Ca^2+^ release via RyR1 turns off, the following applies:

dRnetdt=−uptakeflux (9)

Given that *d*[*Ca*^2+^]_*cyto*_/*dt* at the start of the *off* is ~ *Δ*[Ca^2+^]_cyto_ / τ_cyto_ (where the numerator is the increase in [Ca^2+^]_cyto_ from the resting level) and *dR*_*net*_/*dt* is $∼R_{T}/\tau_{R_{\mathrm{net}}}$, the buffering power of the cytosol is solved as

$$B_{cyto}=\frac{R_{T}\times \tau_{cyto}}{\Delta {\left[Ca^{2+}\right]}_{cyto}\times \tau_{R_{net}}}$$ (10)

With the values presently calculated for WT, *R*_*T*_ = 1655 μM, *Δ*[Ca^2+^]_cyto_ = 0.5 μM, τ_cyto_ = 0.53 s and $\tau_{R_{net}}$ = 3.79 s, *B*_*cyto*_ = 460 (dimensionless, because *R* has dimensions of concentration, see Methods).

The calculated buffering power of the dominant cytosolic buffer, BAPTA, at the concentration (assumed) of 2.5 mM, with a conventional *K*_D_ of 5 μM, would be 500 in the limit of [Ca^2+^] tending to 0, and would be reduced slightly in the conditions of working muscle (by a fraction ~ [Ca^2+^]_cyto_/*K*_D_). The evaluation of *B*_*cyto*_ is therefore consistent with the experimental conditions, which make BAPTA, by far, the dominant buffer.

The corresponding calculation in the Tr-null yields *B*_*cyto*_ = 486. It can be concluded that the buffering power did not change substantially in the null, a result expected for a feature that here is largely determined by the extrinsic buffer in the pipette.

## Conclusions

The ablation of triadin causes major changes in the expression of SR proteins. These result in structural alterations and functional effects on both Ca^2+^ homeostasis and dynamics. The main structural difference, a lower volume of TC, can be ascribed to the reduction in Casq1 expression. The functional effects can be attributed to the loss of conformational, largely inhibitory effects of triadin on RyR1 readiness to open, plus the partial loss of Casq1 and changes in other SR proteins. The altered conformational effects promote both resting and stimulated Ca^2+^ permeability of the SR membrane. The new steady state features a lower [Ca^2+^]_SR_ and its usual consequence, higher [Ca^2+^]_cyto_, probably due to activation of SOCE. The loss of Casq1 results in lower storage capability and Ca^2+^ buffering power. Compounded with the reduced [Ca^2+^]_SR_, these changes lower the quantity of releasable Ca^2+^. The diminished Ca^2+^ bound to Casq1 results in a smaller “hump” component in the flux of Ca^2+^ release upon pulse depolarization. Unexpectedly, the peak component of Ca^2+^ release is largely unchanged in Tr-null muscle. This conservation implies an increase in peak Ca^2+^ permeability of the SR junctional membrane, due to a greater *P*_open_ of individual RyR channels or a greater number of activatable channels. The decrease in buffer capacity of the SR, which results from the lower Casq1 content, is reflected in a faster recovery of peak flux after a depleting pulse. The faster Ca^2+^ reuptake into the SR, which results in a greater rate constant of decay of the cytosolic Ca^2+^ transient, is consistent with the increase in SERCA content and contributes to the faster recovery of Ca^2+^ release.

The deficit in Ca^2+^ release is much less than that predicted by the combined losses in Casq1 endowment, [Ca^2+^]_SR_ and functional voltage sensors; the discrepancy highlights the operation of compensatory mechanisms, the nature of which is yet to be established.

## Supporting information

S1 Fig Fiber composition of Tr-null fibers. *A*, representative immunofluorescence images in (*a*) WT and (*b*) Tr-null FDB cross-sections. Fiber type was determined by isoform-specific myosin heavy chain immunostaining. Red: type I; green: type IIa; blue: type IIx. The fiber type composition was calculated by manually identifying and counting single fibers across the cross-section of each FDB muscle. *B*, Box plots compare the distribution of fiber types in Tr-null (type I, 17.1% ± 3.2, in Tr-null, N = 9; vs. 16.5% ± 1.9 in WT, N = 6; p = 0.56; type IIa 46% ± 4.5 in Tr-null, N = 9; vs. 54.2% ± 5.4 in WT, N = 6; p = 0.14; and type IIx, 36.7% in Tr-null, N = 9; vs. 31.5% ± 5.6 in WT, N = 6; p = 0.42). Symbols represent averages of measurement in one cross-section per animal.
(TIF)

S2 Fig Calsequestrin distribution in Tr-null myofibers. *A*(*a*,*b*), images F(x,y) of CFP-Casq1, locating terminal cisternae, and Di-8-Anepps, marking the t-tubular system, in a WT myofiber. Shown are central images from a z-stack, after correction for optical spread. (*e*, *f*) corresponding images of a Tr-null myofiber. (*c*, *g*) merged images. (*d*, *h*) y-averaged profiles of selected areas in insets, which reveal the relative location of the triad components (terminal cisternae in red trace, at two per triad, and t tubules in green). *B*, averaged Casq1 profiles in triads of WT and Tr-null myofibers. The distance labeled “Triad width” evaluates the joint span of the t tubule plus the two TC. *C*, representation of individual measurements of triad width and two levels of averaging. Individual measures from 40 images of 14 WT myofibers and 135 images of 28 Tr-null myofibers from 3 and 5 mice respectively, are represented as vertical histograms. The horizontal segments at bottom of each histogram provide the scale for numbers of replicas at each ordinate value. Averages of image values for individual fibers are represented by symbols; different symbols identify 3 WT and 5 Tr-null individual mice. Box plots represent distribution of individual measurements (Tr-null: 0.45 ± 0.02. WT: 0.56 ± 0.025 μm). p = 0.007 was calculated using a three-level hierarchical analysis.
(TIF)

S3 Fig Uncropped blots. (TIF)
