# Supplementary figures and images for: Quantification of the calcium signaling deficit in muscles devoid of triadin
Source: PLoS One. 2022 Feb 25;17(2):e0264146. doi: 10.1371/journal.pone.0264146 (PMC8880904; doi:10.1371/journal.pone.0264146)

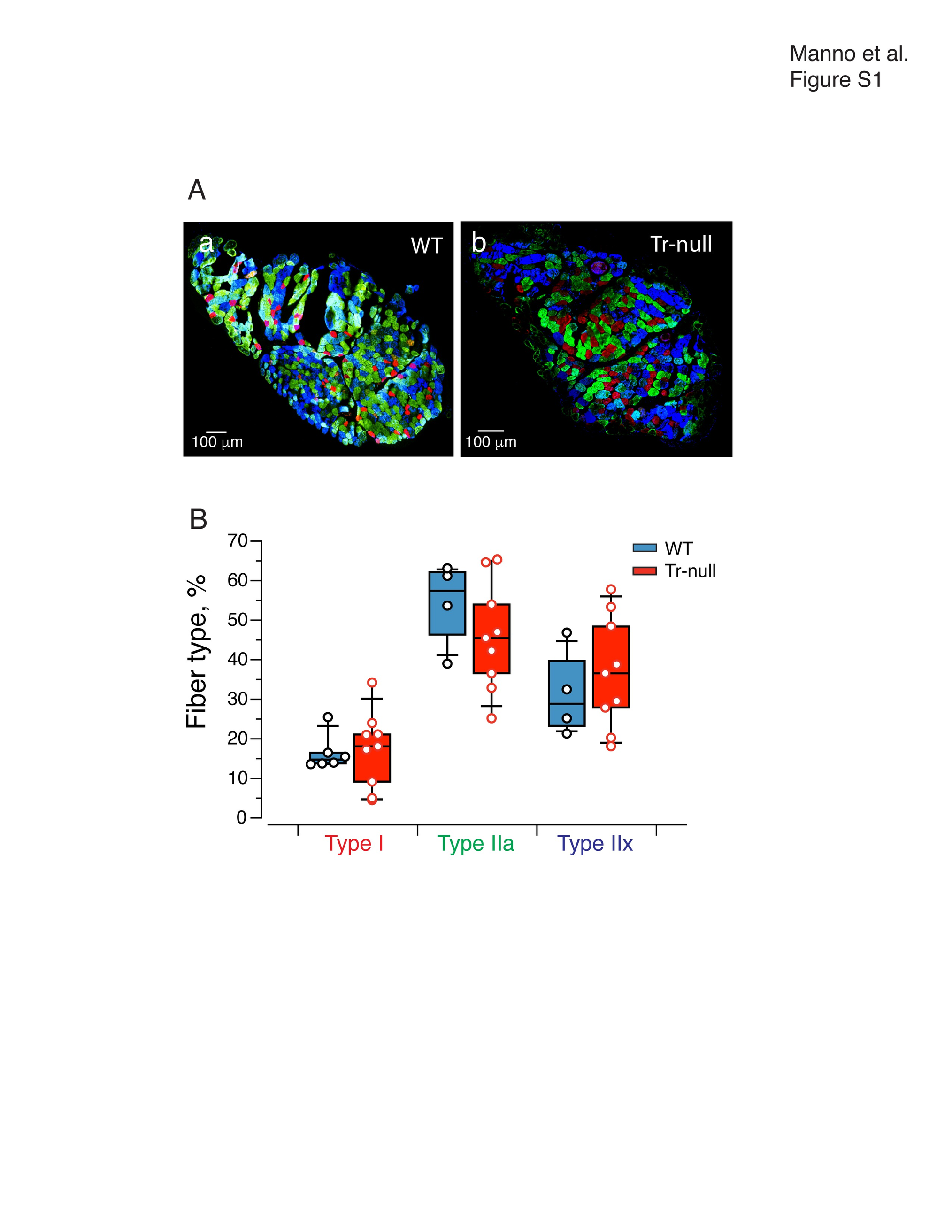

Supplement: S1 Fig — A, representative immunofluorescence images in (a) WT and (b) Tr-null FDB cross-sections. Fiber type was determined by isoform-specific myosin heavy chain immunostaining. Red: type I; green: type IIa; blue: type IIx. The fiber type composition was calculated by manually identifying and counting single fibers across the cross-section of each FDB muscle. B, Box plots compare the distribution of fiber types in Tr-null (type I, 17.1% ± 3.2, in Tr-null, N = 9; vs. 16.5% ± 1.9 in WT, N = 6; p = 0.56; type IIa 46% ± 4.5 in Tr-null, N = 9; vs. 54.2% ± 5.4 in WT, N = 6; p = 0.14; and type IIx, 36.7% in Tr-null, N = 9; vs. 31.5% ± 5.6 in WT, N = 6; p = 0.42). Symbols represent averages of measurement in one cross-section per animal. (TIF) [file pone.0264146.s001.tif]

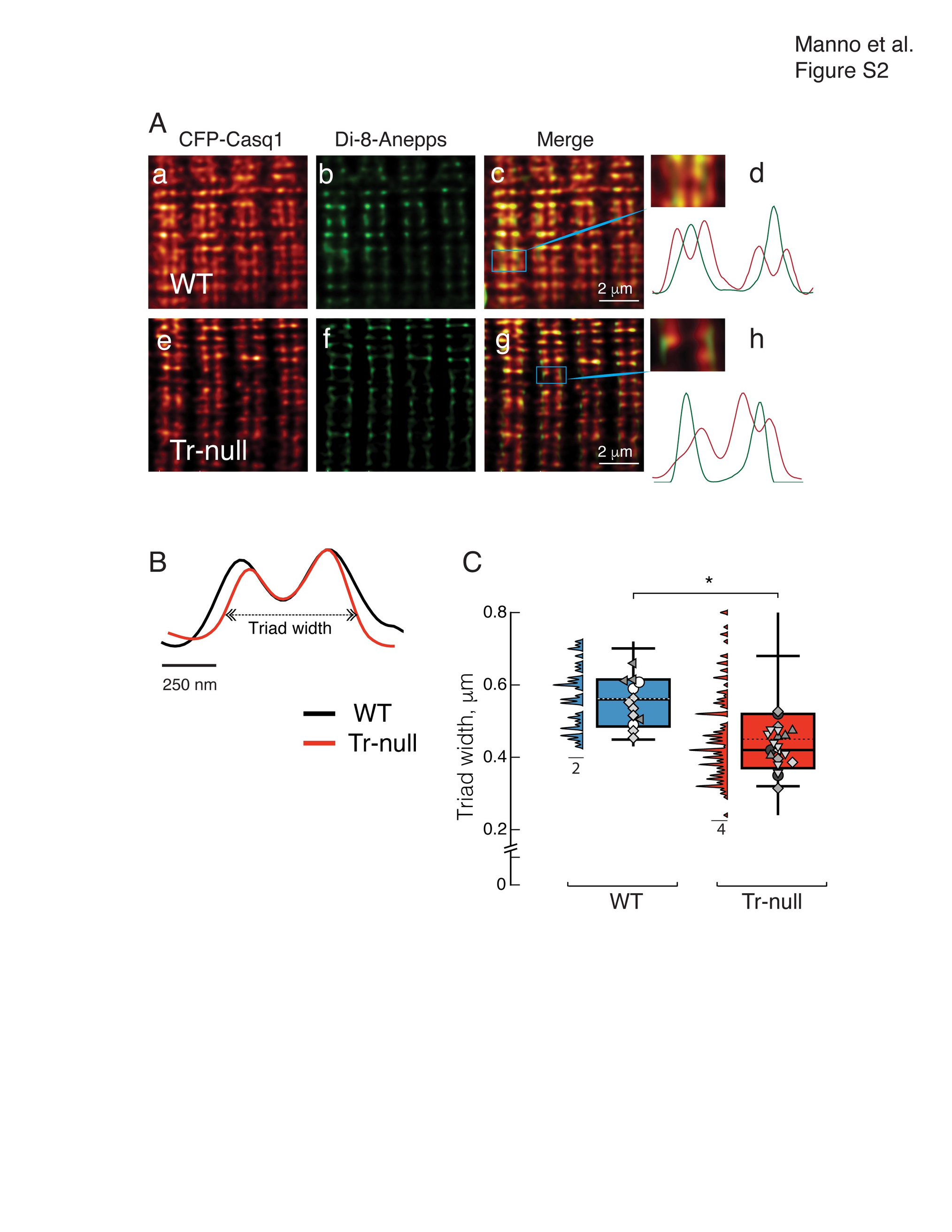

Supplement: S2 Fig — A(a,b), images F(x,y) of CFP-Casq1, locating terminal cisternae, and Di-8-Anepps, marking the t-tubular system, in a WT myofiber. Shown are central images from a z-stack, after correction for optical spread. (e, f) corresponding images of a Tr-null myofiber. (c, g) merged images. (d, h) y-averaged profiles of selected areas in insets, which reveal the relative location of the triad components (terminal cisternae in red trace, at two per triad, and t tubules in green). B, averaged Casq1 profiles in triads of WT and Tr-null myofibers. The distance labeled “Triad width” evaluates the joint span of the t tubule plus the two TC. C, representation of individual measurements of triad width and two levels of averaging. Individual measures from 40 images of 14 WT myofibers and 135 images of 28 Tr-null myofibers from 3 and 5 mice respectively, are represented as vertical histograms. The horizontal segments at bottom of each histogram provide the scale for numbers of replicas at each ordinate value. Averages of image values for individual fibers are represented by symbols; different symbols identify 3 WT and 5 Tr-null individual mice. Box plots represent distribution of individual measurements (Tr-null: 0.45 ± 0.02. WT: 0.56 ± 0.025 μm). p = 0.007 was calculated using a three-level hierarchical analysis. (TIF) [file pone.0264146.s002.tif]

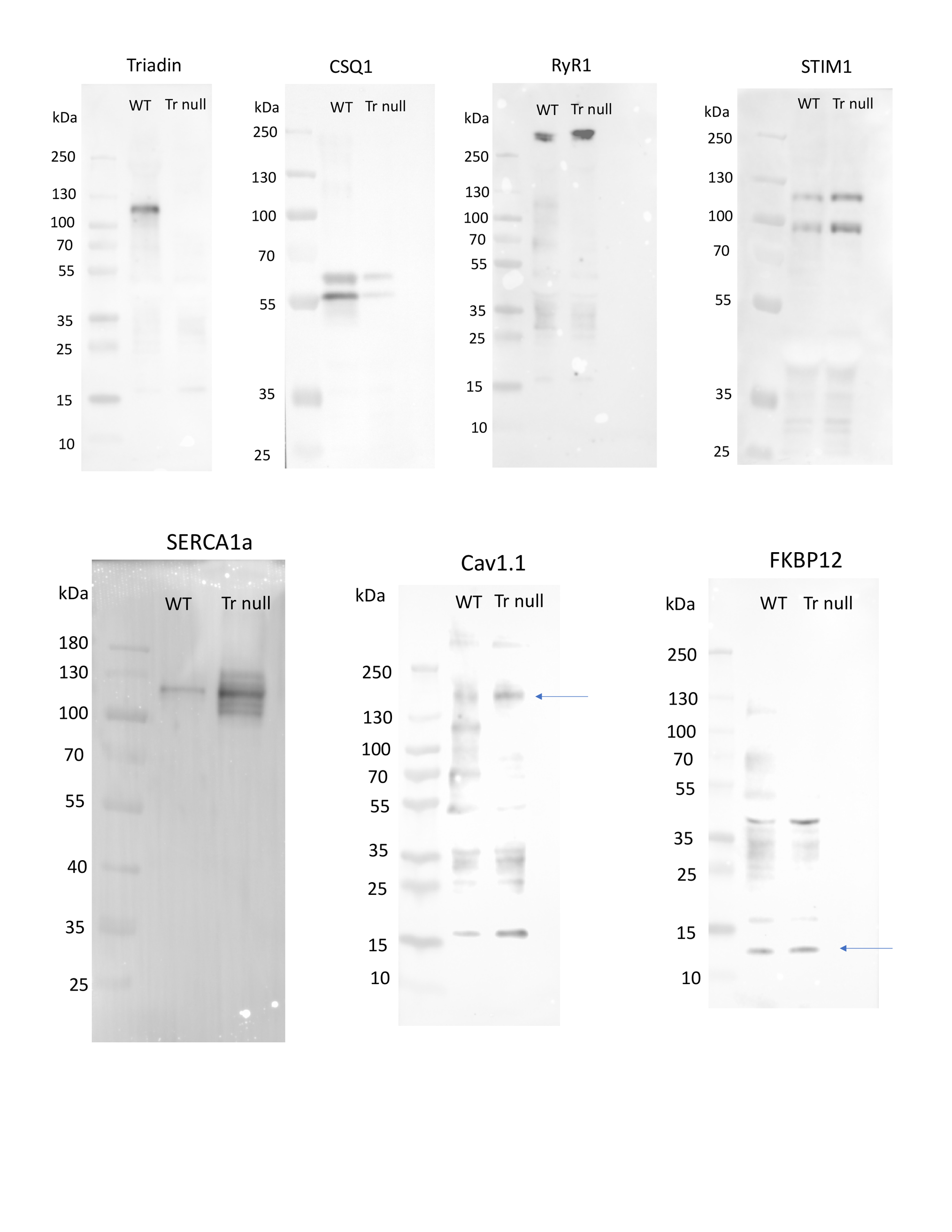

Supplement: S3 Fig — (TIF) [file pone.0264146.s003.tif]
